# Premature cell senescence promotes vascular smooth muscle cell phenotypic modulation and resistance to re-differentiation

**DOI:** 10.1093/cvr/cvaf102

**Published:** 2025-06-10

**Authors:** Anuradha Kaistha, Sebnem Oc, Abel Martin Garrido, James C K Taylor, Maria Imaz, Matthew D Worssam, Anna Uryga, Mandy Grootaert, Kirsty Foote, Alison Finigan, Nichola Figg, Helle F Jørgensen, Martin Bennett

**Affiliations:** Section of Cardiorespiratory Medicine, University of Cambridge, Victor Phillip Dahdaleh Heart & Lung Research Institute, Papworth Road, Cambridge Biomedical Campus, Cambridge CB2 0BB, UK; Section of Cardiorespiratory Medicine, University of Cambridge, Victor Phillip Dahdaleh Heart & Lung Research Institute, Papworth Road, Cambridge Biomedical Campus, Cambridge CB2 0BB, UK; Section of Cardiorespiratory Medicine, University of Cambridge, Victor Phillip Dahdaleh Heart & Lung Research Institute, Papworth Road, Cambridge Biomedical Campus, Cambridge CB2 0BB, UK; Section of Cardiorespiratory Medicine, University of Cambridge, Victor Phillip Dahdaleh Heart & Lung Research Institute, Papworth Road, Cambridge Biomedical Campus, Cambridge CB2 0BB, UK; Section of Cardiorespiratory Medicine, University of Cambridge, Victor Phillip Dahdaleh Heart & Lung Research Institute, Papworth Road, Cambridge Biomedical Campus, Cambridge CB2 0BB, UK; Section of Cardiorespiratory Medicine, University of Cambridge, Victor Phillip Dahdaleh Heart & Lung Research Institute, Papworth Road, Cambridge Biomedical Campus, Cambridge CB2 0BB, UK; Section of Cardiorespiratory Medicine, University of Cambridge, Victor Phillip Dahdaleh Heart & Lung Research Institute, Papworth Road, Cambridge Biomedical Campus, Cambridge CB2 0BB, UK; Section of Cardiorespiratory Medicine, University of Cambridge, Victor Phillip Dahdaleh Heart & Lung Research Institute, Papworth Road, Cambridge Biomedical Campus, Cambridge CB2 0BB, UK; Section of Cardiorespiratory Medicine, University of Cambridge, Victor Phillip Dahdaleh Heart & Lung Research Institute, Papworth Road, Cambridge Biomedical Campus, Cambridge CB2 0BB, UK; Section of Cardiorespiratory Medicine, University of Cambridge, Victor Phillip Dahdaleh Heart & Lung Research Institute, Papworth Road, Cambridge Biomedical Campus, Cambridge CB2 0BB, UK; Section of Cardiorespiratory Medicine, University of Cambridge, Victor Phillip Dahdaleh Heart & Lung Research Institute, Papworth Road, Cambridge Biomedical Campus, Cambridge CB2 0BB, UK; Section of Cardiorespiratory Medicine, University of Cambridge, Victor Phillip Dahdaleh Heart & Lung Research Institute, Papworth Road, Cambridge Biomedical Campus, Cambridge CB2 0BB, UK; Section of Cardiorespiratory Medicine, University of Cambridge, Victor Phillip Dahdaleh Heart & Lung Research Institute, Papworth Road, Cambridge Biomedical Campus, Cambridge CB2 0BB, UK

**Keywords:** Atherosclerosis, smooth muscle, senescence, DNA damage

## Abstract

**Aims:**

Human atherosclerotic plaque cells display DNA damage that if left unrepaired can promote premature cell senescence. Vascular smooth muscle cells (VSMCs) predisposed to senescence promote atherogenesis and features of unstable plaques and increase neointima formation after injury. However, how premature VSMC senescence promotes vascular disease and its effects on VSMC phenotype are unknown.

**Methods and results:**

Bulk RNA-seq of primary human VSMCs identified 126 significantly up- or down-regulated genes after both DNA damage-induced (D + R) or replicative senescence (RS). Up-regulated genes included senescence markers *CDKN2A* (p16) and *ICAM1* and genes expressed by phenotypically modulated de-differentiated/’fibromyocytic’ VSMCs [osteoprotegerin (*TNFRSF11B*), fibromodulin (*FMOD)*] as well as transmembrane protein 178B (*TMEM178B*) and secreted frizzle-related protein 4 (*SFRP4*). Mouse VSMCs also up-regulated genes associated with de-differentiated VSMC phenotype, *Tmem178b* and *Sfrp4* after D + R. Single-cell RNA-sequencing of lineage-traced VSMCs in mouse plaques or human plaques showed that VSMCs expressing *Cdkn2a* had lower contractile marker expression and higher expression of de-differentiated VSMC markers. Mice expressing a VSMC-restricted mutant telomere protein (TRF2^T188A^) that induces premature senescence showed increased atherosclerosis, expression of multiple de-differentiation genes in plaques and after injury, and differential regulation of pathways associated with extracellular matrix organization, inflammation and Transforming Growth Factor-β (Tgfb). *Trf2^T188A^* VSMCs were more resistant to re-differentiation and had dysregulated Tgfb signalling at multiple levels with down-regulated ligands, receptors, and coactivators and up-regulated co-repressor expression. *Trf2^T188A^* VSMCs also showed cytosolic DNA and activation of the STING–TBK1–IRF3 pathway that suppressed Tgfb signalling. Silencing *IRF3* restored expression of Tgfb pathway components and VSMC contractile markers after TGFb administration.

**Conclusion:**

DNA damage and senescence induce genes associated with de-differentiated/fibromyocytic VSMCs, and persistence of these cells *in vivo.* Failure of senescent VSMCs to re-express contractile markers during re-differentiation suggests that VSMC senescence may promote atherosclerosis and neointima formation in part by inhibiting their re-differentiation.

## Graphical Abstract

**Graphical Abstract:**
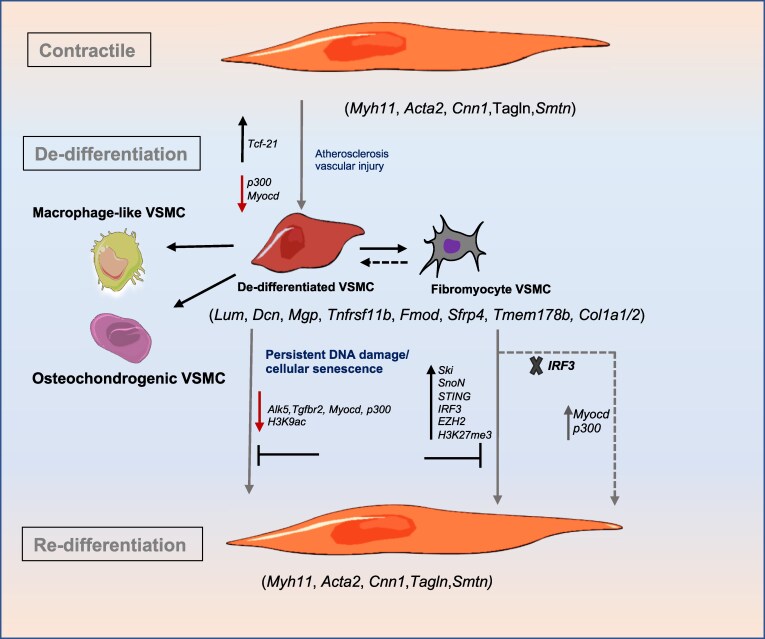
Senescence affects phenotype of neointimal VSMCs *in vivo.* Model of how persistent DNA damage/cell senescence affects VSMC phenotype. VSMCs undergo de-differentiation in culture or *in vivo* (e.g. in atherosclerosis or injury) regulated by factors such as *Myocd* and *Tcf21*. These de-differentiated VSMCs can undergo further phenotypic modulation, for example to fibromyocytes, macrophage-like cells, or osteochondrogenic cells, which express some de-differentiation markers but also some more cell state-specific markers, such as *Fmod*, *Sfrp4* and *Tnfrsf11b* for fibromyocytes. Both de-differentiated and fibromyocyte VSMCs may re-differentiate to a contractile phenotype. However, senescence results in maintenance of a de-differentiated/fibromyocyte state, potentially due to cytosolic DNA-mediated *STING/TBK1/IRF3* upregulation that suppresses multiple components of Tgfb signalling and contractile genes. Silencing IRF3 partly restores TGFb-mediated re-differentiation of VSMCs.

**Time of primary review: 40 days**

## 1. Introduction

Vascular smooth muscle cells (VSMCs) in human atherosclerotic plaques display a wide range of different types of DNA damage, including double-strand breaks,^1^ telomere damage,^2,3^ and oxidative DNA damage.^4^ Telomeres are particularly sensitive to DNA damage, in part because they are not targeted by most general DNA repair pathways, and VSMCs in human plaques show reduced telomere length and lower expression and binding of specific protective telomere proteins such as telomere repeat binding factor 2 (TRF2).^2,3^ VSMC telomere damage occurs during replication or after stimuli such as oxidant stress,^2^ and progressive DNA damage results in premature senescence characterized by growth arrest, activation of a persistent DNA damage response (DDR), expression of different senescence marker genes, and secretion of a panel of pro-inflammatory cytokines (the senescence-associated secretory phenotype—SASP). Cell senescence has been described in atherosclerosis and after vascular injury, including in endothelial cells and VSMCs (e.g.^5–9^). Furthermore, a number of studies have shown that VSMCs predisposed to senescence promote atherogenesis and features of plaque vulnerability, including reduced fibrous caps and increased neointima formation after injury,^3,4,10,11^ but the underlying mechanisms are unclear and may be multiple. For example, senescent VSMCs show loss of proliferation and reduced migration and express genes with roles in inflammation, tissue remodelling and vascular calcification.^12,13^

DNA damage promotes expression of pro-inflammatory cytokines in VSMCs,^4,10^ similar to those seen after VSMC de-differentiation,^14^ but does not affect the number of VSMC-derived cells in the neointima or VSMC clonality after injury,^10^ suggesting that primary defects in initial de-differentiation, proliferation, or migration are not responsible. However, how DNA damage affects VSMC phenotypic modulation is unknown, particularly in those cells that retain or regain contractile markers after injury or in fibrous caps in atherosclerosis. VSMCs in culture, atherosclerosis and after injury exhibit a range of different phenotypes, described as ‘contractile’, ‘synthetic’, ‘adipocyte-like’, ‘foam cell’, ‘macrophage-like’, ‘osteocyte-like’, and ‘chondrocyte-like’ cells, with relative proportions differing according to context (reviewed in.^15^) VSMC lineage tracing studies combined with single-cell RNA-sequencing (scRNA-seq) have described the transcriptomic heterogeneity of VSMCs underlying these phenotypes, and also revealed similarity of transcriptomic states between mouse and human atherosclerosis (e.g.^16,17–19^) These studies also identified a transitional de-differentiated multipotent population known as ‘stem cell, endothelial cell and monocyte’ (SEM) cells^18^ or ‘pioneer’ cells,^17^ that may represent an intermediate VSMC phenotypic switching state.^18,20^ Specific populations of these cells, for example those expressing the progenitor cell marker Stem cell antigen 1 (SCA1), may then generate many other different phenotypes,^21^ including ‘fibromyocytes’ that may contribute to VSMCs that express contractile markers and overlie plaque or neointima.^19^ However, VSMC-derived plaque cells represent a continuum with relative rather than absolute changes in the expression of specific genes, and both regulation and markers of specific subsets may differ between human and mouse VSMCs.

We examined the VSMC senescence and phenotype markers gene using two models of senescence of human VSMCs, scRNA-seq data from human and mouse atherosclerotic plaques, and mice with VSMC telomere damage with atherosclerosis and after injury. VSMC senescence up-regulated multiple de-differentiation/fibromyocytic markers in human and mouse VSMCs. VSMCs predisposed to undergo senescence expressed higher levels of de-differentiation markers *in vivo* and were more resistant to re-differentiation *in vitro*. We suggest that VSMC senescence may promote atherosclerosis or neointima formation in part by retaining VSMCs in a de-differentiated phenotype.

## 2. Methods

### 2.1 Human tissue

Human tissue was obtained under written informed consent using protocols approved by Cambridge or Huntingdon Research Ethical Committees, conforming to the principles outlined in Declaration of Helsinki. Carotid endartectomy samples were obtained from Royal Papworth Hospital tissue bank with ethical committee approval. Primary human aortic VSMCs were isolated from explants as described in Supplementary Material online.

### 2.2 Animal experiments

Animal experiments were performed under Animals (Scientific Procedures) Act 1986 Amendment Regulations 2012 and approved by Cambridge Animal Welfare and Ethical Review Body (AWERB). Mice were anaesthetized with 2.5% inhalable isoflurane (maintained at 1.5%), monitoring respiratory and heart rates, muscle tone and reflexes, and euthanized by CO_2_ overdose. Male and female C56BL/6J mice were used for all experiments, except *Myh11-CreER^t2^/Confetti/ApoE^−/−^* mice where only males expressed the tamoxifen-inducible CRE. Primary mouse aortic VSMCs were isolated, and EdU and SAβG activity assays were performed as described in Supplementary Material online.

### 2.3 Molecular analysis

RNA isolation, cDNA preparation, qPCR primers and quantification conditions are described in Supplementary Material online and Supplementary material online, *Table S1*.

### 2.4 Western blots

Western blots and antibodies used are as described in Supplementary Material online and Supplementary material online, *Table S2*.

### 2.5 Confocal microscopy

Immunofluorescence and imaging of formalin-fixed, paraffin-embedded human carotid endarterectomy sections, control and Trf2^T188A^ VSMCs, ligated carotid artery sections and quantification of confetti-positive fibrous cap cells were performed as described in Supplementary Material online.

### 2.6 Immunohistochemistry

Bright-field imaging was performed as described in Supplementary Material online.

### 2.7 RNA-seq

Bulk RNA-seq was performed on human primary aortic VSMC cultures treated with Doxorubicin for 1d + 21d recovery (D + R), replicative senescence (RS), or Dox 1d, or on control 1d replicating cells. Sample preparation, sequencing and data processing were performed as described previously^10^ and in Supplementary Material online.

Atherosclerosis scRNA-seq profiles from human coronary lesions^19^ (GSE131778) were analysed as described in Supplementary Material online^22^ and murine VSMC-derived plaque and medial cells isolated from fat-fed *Myh11-cre^ERT2^/Rosa 26-Confetti/ApoE^−/−^* mice^16^ (GSE117963) were clustered and gene expression analysis performed as previously described.^19,21^

For scRNA-seq profiles from *Trf2^T188A^/ApoE^−/−^* vs. *ApoE^−/−^* mice, plaques were micro-dissected from the aorta (root and arch) and carotid arteries and underwent enzymatic digestion. Adventitia and non-remodelled arterial sections were removed before enzymatic digestion from ligated carotid arteries. Single-cell suspensions were analysed using a Chromium system (10 × Genomics). Raw sequencing reads were aligned to the GRCm38 mouse genome using the 10 × Genomics Cell Ranger pipeline (v.7.0.0 and v.6.1.2, respectively, GSE210406). Data analysis was performed as described in Supplementary Material online.

### 2.8 Mouse carotid artery ligation

Carotid artery ligation was performed as described previously;^10^ 4- to 5-m-old male C57Bl6/J *Sm22a-TRF2^T188A^* or wild-type littermate control mice backcrossed >10 × received pre-operative buprenorphine (0.1 mg/kg subcutaneously) and inhalable isoflurane. The left common carotid artery (LCCA) was ligated just below the bifurcation with a 6–0 silk suture. Following 28 days of recovery, the LCCA was collected and processed as described for scRNA-seq^16^ or confocal microscopy.^10^

### 2.9 Mouse atherosclerosis studies

Transgenic mice generation, genotyping protocols, power calculation and animal randomization procedures were as described previously.^16^ C57Bl6/J *Sm22a-Trf2^T188A^/ApoE^−/−^* or wild-type littermate *ApoE^−/−^* mice were crossed with *Myh11-CreER^t2^/Confetti/ApoE^−/−^* mice to generate *Myh11-CreER^t2^/Confetti/Sm22a-Trf2^T188A^*/*ApoE^−/−^ (Trf2^T188A^/ApoE^−/−^*) or *Myh11-CreER^t2^/Confetti/ApoE^−/−^ (ApoE^−/−^*) mice, and 10 injections of 100 µL of 1 mg/mL tamoxifen (Sigma-Aldrich) administered over 2 weeks at 6 weeks of age. Following one-week rest, mice were administered high fat diet (HFD, 829-100, Special Diet Services, UK, 21% total fat, 0.2% cholesterol, 0% sodium cholate) for 14weeks. Blood pressure, serum lipids and cytokines were analysed as described in Supplementary Material online.

### 2.10 Re-differentiation and exogenous DNA transfection protocol

Control or *Trf2^T188A^* VSMCs were seeded in DMEM + 10% serum and 1% v/v L-glutamine/penicillin/streptomycin solution, attached for 24 h, washed with warm PBS and serum-free medium, and then serum-starved for 4–6 h. Cells were then treated with recombinant TGFb1 (10 ng/mL, R&D Systems 240-B-002) or vehicle for 1, 24, or 48 h in DMEM containing 0.5% serum or left in 2.5% serum medium overnight before administering 50 nM Rapamycin (Millipore Sigma- 553211) for 24 h in DMEM + 2.5% serum. For exogenous DNA transfection experiments, cells were transfected with 1 ug/mL Herring testis (HT) dsDNA (Millipore Sigma-D6898) for 24 h using Lipofectamine™ 3000 (Thermo Fisher Scientific) or Lipofectamine alone, and then administered TGFb1 for 48 h.

### 2.11 siRNA transfection

For *IRF3* silencing, control and *Trf2^T118A^* VSMCs were transfected with 50 nM of SMARTPool *Irf3* (L-041095-00-0005, Horizon) or non-targeting control siRNAs (D-001810-10-05, Horizon) with Lipofectamine RNAiMAX (Thermo Fisher Scientific), cultured for 48 h, and administered recombinant TGFb1 or vehicle for 48 h.

### 2.12 Statistics

RNA-seq data statistical analysis is described in Supplementary Material online. GraphPad Prism 9.00 (GraphPad Software Inc.) was used for other statistical analysis. After outlier identification (ROUT method *Q* = 1%) and Shapiro–Wilk test for normality distribution, statistical significance was determined by unpaired Student’s *t*-test for normally distributed data with similar SDs, or Welch’s *t*-test without similar SDs. Mann–Whitney test was used for data that were not normally distributed. One-way ANOVA followed by Tukey’s or Bonnferroni’s multiple comparison was used for comparisons of more than two groups. Kruskal–Wallis H-test with Dunn’s multiple comparisons test was used if data were not normally distributed, as detailed in figure legends. Experimental reproducibility was achieved by the following actions: (i) the operator conducting carotid ligation surgery was blinded to mouse genotype, and (ii) tissue sections were analysed blindly. Confounding factors were reduced by (i) wild-type littermates from *Sm22a-Trf2^T188A^* mice were used as controls, (ii) both genotypes were housed in the same cages during colony expansion and experiments, and (iii) underwent carotid ligation surgery and tissue collection on the same day. All data are shown in dot plots to demonstrate data distribution and represent individual biological not technical replicates. Data are presented as mean ± SD and statistically significant difference considered as *P* < 0.05.

## 3. Results

### 3.1 Models of human VSMC senescence

We examined gene expression in two models of human VSMC senescence characterized by persistent telomere DNA damage. Human VSMCs treated with doxorubicin for 24-h manifest widespread DNA damage; cells normalize global DNA damage markers after 21-day recovery, but telomere damage persists with reduced TRF2 expression.^10^ Similarly, cultured human VSMCs progressively shorten telomeres and ultimately enter replicative senescence (RS). We have previously shown that Dox 1d + 21d treatment or RS results in <10% EdU incorporation over 48 h, no increase in cell number over 14d, and >70% senescence-associated beta-galactosidase’ (SAβG) expression.^23^ These protocols also reduced Lamin B1 and increased p16 expression, established markers of senescence, but not p53 or p21 DNA damage markers (see Supplementary Material online, *Figure S1A* and *B*).

### 3.2 VSMC senescence induces genes associated with a de-differentiated ‘fibromyocyte’ phenotype

**Figure 1:**
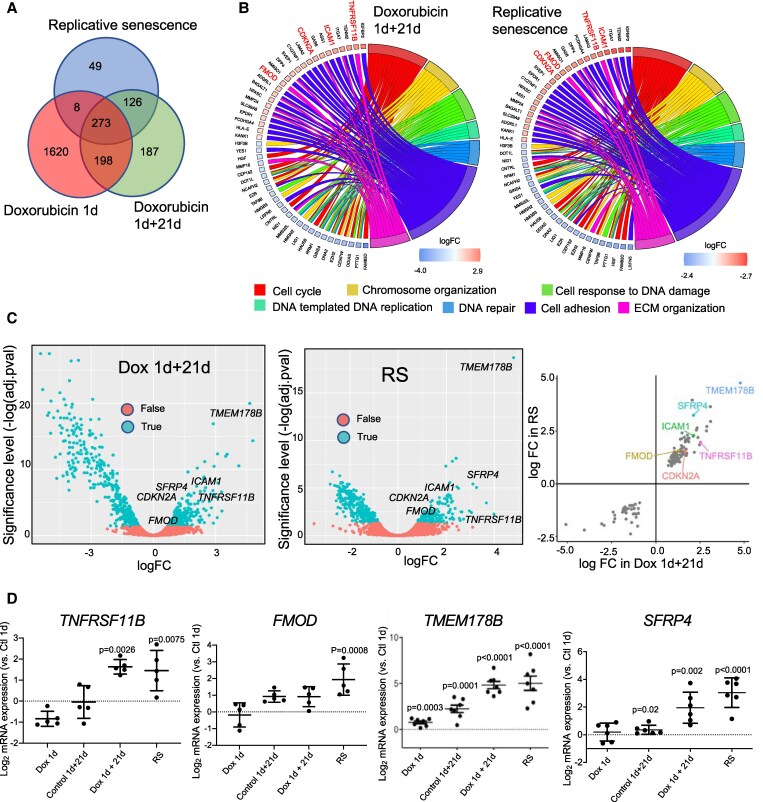
Replicative and stress-induced premature senescence of human vascular smooth muscle cells induce genes associated with a de-differentiated phenotype, TMEM178B and SFRP4 . (*A*) Venn diagram of RNA-seq data showing numbers of genes unique to RS, Dox 1d + 21d, or Dox 1d, or genes common to two or three groups. (*B*) Chord plots for selected Gene ontology (GO) terms enriched within the up-regulated or down-regulated genes (*P*-adjusted < 0.05) in cells following Dox 1 + 21d treatment or replicative senescence (RS). (*C*) Volcano plots (left and middle) showing differential gene expression in human VSMCs after treatment with Dox 1d + 21d or RS compared to Control 1d, or scatter plot (right). *TNFRSF11B, FMOD, TMEM178B*, *SFRP4, ICAM1,* and *CDKN2A* (p16) are marked. False/True indicates False Discovery Rate (FDR)-adjusted *P*-value < 0.05, Fold change (FC) relative to control 1d. (*D*) Transcript levels of *TNFRSF11B, FMOD, TMEM178B,* and *SFRP4* measured by RT-QPCR in human VSMC samples shown relative to Control [1d (*n* = 5–7 human VSMC isolates)]. Dot plots represent individual samples, mean and SD. 1-Way ANOVA.

Human primary passage 4–5 ascending aortic VSMCs from four donors (two male, two female; average age, 65years) underwent Dox 1d, Dox 1d + 21d, or RS treatments, followed by bulk RNA-seq to compare gene expression against Control 1d (replicating) cells. 126 genes showed statistically significant increased or decreased expression after both Dox 1d + 21d and RS compared with Control 1d replicating cells, but were not affected by acute DNA damage (Dox 1d) (*Figure 1A,*Supplementary Material online, *Table S3*). Gene Ontology (GO) analysis (*P*-adjusted < 0.05) showed enrichment of pathways associated with extracellular matrix (ECM) organization and stress responses after Dox 1d + 21d and RS. Up-regulated genes were also significantly associated with cell adhesion while down-regulated genes were significantly associated with cell cycle, DNA replication, response to DNA damage, and DNA repair, consistent with senescence (*Figure 1B*, Supplementary Material online, *Table S4*). Dox 1d + 21d and RS up-regulated some genes previously associated with VSMC or fibroblast senescence, including *p16/CDKN2A* (LogFC 1.5–1.6), intercellular adhesion molecule 1 (*ICAM1*, LogFC 2.1–2.3), transmembrane protein 178B (*TMEM178B*) and secreted frizzle-related protein 4 (*SFRP4*)^10,24,25^ (*Figure 1C*, Supplementary Material online, *Table S3*), but importantly several genes associated with a de-differentiated ‘fibromyocyte’ VSMC subset,^19^ including tumour necrosis factor receptor superfamily member 11b (*TNFRSF11B*)/osteoprotegerin (LogFC 2.0–2.5) and fibromodulin (*FMOD*, LogFC 1.3–1.6) (*Figure 1C*, Supplementary Material online, *Table S3*), which have been implicated in fibrous cap formation,^19^ suggesting that senescence may affect VSMC phenotypic switching.

### 3.3 Validation and expression of SAGs in human atherosclerotic plaques

*TNFRSF11B*, *FMOD,* and *SFRP4* were included for further study as they have been identified as genes expressed by fibromyocytic VSMCs (*TNFRSF11B, FMOD*) and/or have a role in atherosclerosis (*TNFRSF11B*,^26,27,28^*FMOD*,^29^*SFRP*4,^30,31^) or up-regulated in fibroblast senescence (*TMEM178B*, *SFRP4*).^25^ 5–7 additional human VSMC isolates were used to validate changes in these genes in senescence. RS increased both *TNFRSF11B* and *FMOD* mRNA levels, and Dox 1d + 21d also increased *TNFRSF11B*. Although *TMEM178B* and *SFRP4* expression were slightly increased in control (1d + 21d) cells, most likely reflecting continued culture, both transcripts increased very significantly in Dox 1d + 21d and RS vs. Control 1d cells (*Figure 1D*). These data indicate that *TNFRSF11B, FMOD*, *TMEM178B*, and *SFRP4* are ‘senescence-associated genes’ (SAGs) in human VSMCs, and that senescence may promote a de-differentiated/fibromyocytic phenotype in VSMCs.

**Figure 2:**
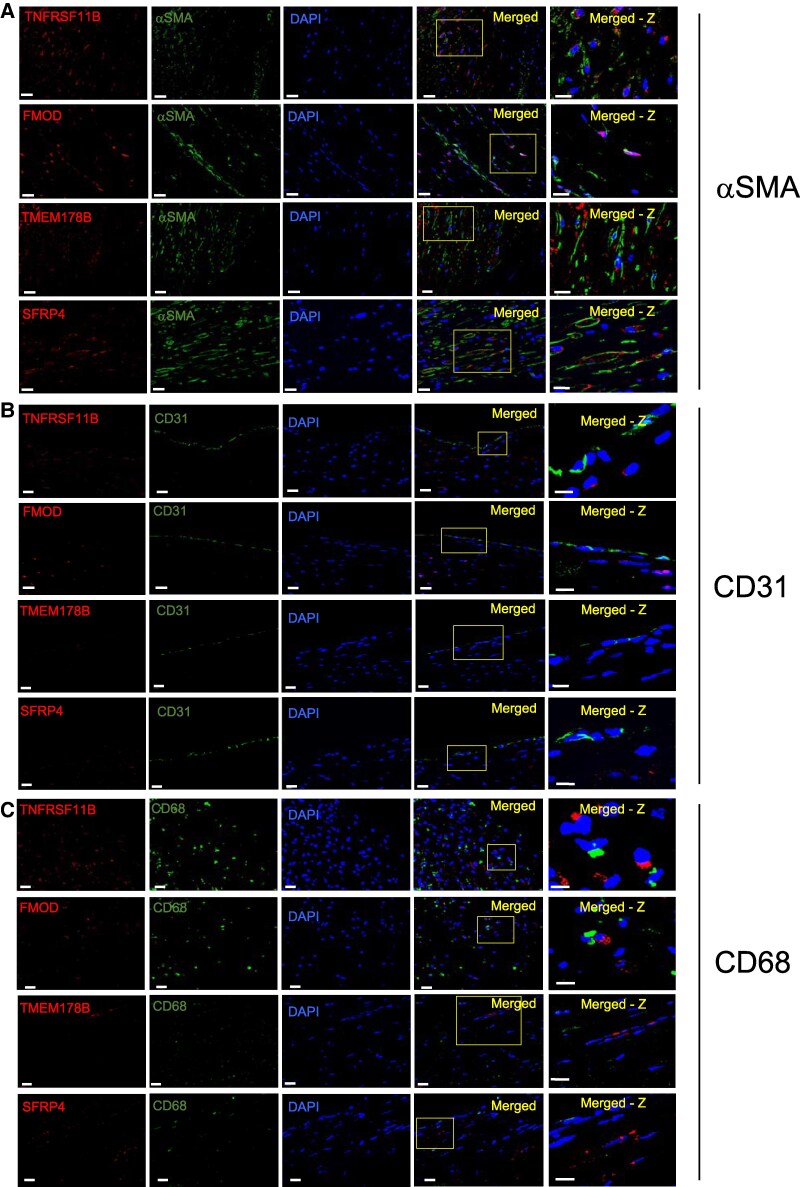
De-differentiated phenotype genes, TMEM178B, and SFRP4 are expressed by VSMCs in atherosclerosis. Confocal microscopy images of human plaques co-stained for TNFRSF11B, FMOD, TMEM178B, or SFRP4, together with aSMA/ACTA2 (*A*), CD31 (*B*), or CD68 (*C*) with 4′,6-diamidino-2-phenylindole (DAPI) and merged image with Z-series reconstruction. Scale bars = 10μm and 5μm (merged *Z*-stack).

VSMCs expressing senescence markers are located particularly in the fibrous cap in human plaques,^2,3^ and although TNFRSF11B, FMOD, and SFRP4 proteins are all expressed by VSMCs and fibroblasts in normal human vessels, human protein atlas data indicate that endothelial cells can also express TMEM178B (https://www.proteinatlas.org/). Confocal microscopy including Z-series reconstruction showed that all four genes co-localized with αSMA/ACTA2 in human plaques, particularly in fibrous caps, and were not found in luminal ECs (*Figure 2A–C*). No signals were observed with relevant isotype control antibodies or no antibodies to exclude autofluorescence (see Supplementary Material online, *Figure S2)*.

### 3.4 De-differentiation genes, Tmem178b, and Sfrp4 are expressed by mouse VSMCs undergoing senescence

**Figure 3:**
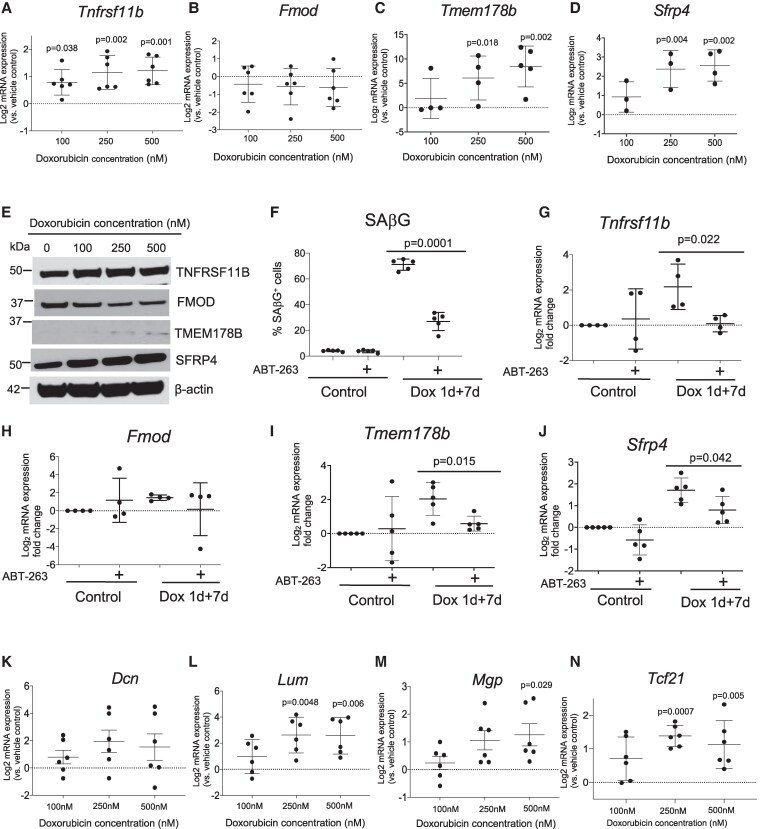
VSMC de-differentiation genes, *Tmem178b*, and *Sfrp4* are induced in mouse VSMC senescence. (*A–D*) Transcript levels of *Tnfrsf11b, Fmod, Tmem178b, and Sfrp4* measured by RT-QPCR in mouse VSMCs treated with increasing concentrations of Dox (1d + 7d) relative to vehicle control. *n* = 3–6, one-way ANOVA. (*E*) Western blot for TNFRSF11B, FMOD, TMEM178B, and SFRP4 for cells in (*A–D*). (*F*) % SAβG^+^ mouse VSMCs after control or Dox 1d + 7d treatment, ± ABT-263 for 48 h; *n* = 5, unpaired *t*-test (*G–J*) Fold change in mRNA expression ± ABT-263 treatment against the housekeeping gene *Hmbs* for *Tnfrsf11b* (*G*), *Fmod* (*H*)*, Tmem178b* (*I*), and *Sfrp4* (*J*); *n* = 4–5, unpaired *t*-test. (*K–N*) Transcript levels of *Dcn, Lum, Mgp,* and *Tcf21* measured by RT-QPCR in mouse VSMCs treated with increasing concentrations of Dox (1d + 7d) relative to vehicle control. n = 6, one-Way ANOVA.

Senescence-associated genes (SAGs) vary with species, inducer and cell type and VSMC phenotype markers are often inconsistent across species; we therefore examined SAG expression in mouse VSMCs undergoing senescence. Primary mouse VSMCs treated with increasing Dox concentrations for 1d + 7d recovery (Dox 1 + 7d) show <6% EdU^+^ and >85% %SAβG^+^ cells,^23^ and reduced Lamin B1 expression but increased p16, p53 and p21 (see Supplementary Material online, *Figure S3A)* consistent with senescence*. Tnfrsf11b, Tmem178b*, and *Sfrp4* mRNA and protein expression showed a significant concentration-dependent increase after Dox 1d + 7d vs. vehicle control (*Figure 3A–E*, Supplementary Material online, *Figure S3B*), suggesting that like human VSMCs, DNA damage-induced senescence promotes a de-differentiated/fibromyocytic phenotype in mouse VSMCs.

Increased gene expression in senescent cells can be induced by senescence itself or paracrine effects of multiple SASP cytokines.^10^ However, although the pro-inflammatory stimulus (lipopolysaccharide) administered for 1d or 1d + 7d recovery significantly increased *Il6* mRNA, *Cdkn2a/p16, Tnfrsf11b*, *Tmem178b*, *Sfrp4*, and *Fmod* mRNA expression were unchanged (see Supplementary Material online, *Figure S4*), suggesting that their induction is not caused by external pro-inflammatory signals. To examine whether these genes are preferentially expressed in senescent VSMCs, we removed these cells using the senolytic ABT-263.^8,32,33^ SAβG^+^ VSMCs were very infrequent in control cultures and ABT-263 did not affect *Tnfrsf11b*, *Fmod, Tmem178b,* and *Sfrp4* expression; however, ABT-263 significantly reduced %SAβG^+^ VSMCs (*Figure 3F*, Supplementary material online, *Figure S5*), and *Tnfrsf11b, Tmem178b*, and *Sfrp4* mRNA expression in Dox 1d + 7d samples (*Figure 3G–J*). Finally, we examined a range of additional ‘de-differentiation’ genes in mouse VSMCs after Dox 1d + 7d treatment. *Lum*, *Mgp,* and *Tcf21* were also significantly induced by Dox 1d + 7d (*Figure 3K–N*), together indicating that senescence of mouse VSMCs also upregulates de-differentiation/fibromyocytic genes.

### 3.5 Expression of senescence genes in VSMCs in human and mouse atherosclerosis

To assess whether these SAGs are also expressed in senescent VSMCs *in vivo*, we examined a previously published human coronary plaque scRNA-seq dataset from 4 separate donors^19^ (see Supplementary Material online, *Figure S6A*–*D*). Typical contractile VSMC markers (*MYH11*, *ACTA2, CNN1*, and *TAGLN*) were highly expressed in the (contractile) VSMC cluster and reduced in the Fibromyocyte cluster (see Supplementary Material online, *Figure S6A*–*C)*. *TNFRSF11B*, *FMOD,* and *SFRP4* were mostly expressed in the Fibromyocyte population, which also expressed *CDKN2A/p16*, *MGP*, *FN1*, *COL1A1,* and *COL3A1*, while *LUM* and *DCN* were expressed in both Fibroblasts and Fibromyocyte clusters. Correlation analysis confirmed that *p16/CDKN2A* expression positively correlates with *TNFRSF11B, FMOD* and *SFRP4* (see Supplementary Material online, *Figure S6D*) but not the VSMC contractile markers. *TMEM178B* was not detected. We also examined a scRNA-seq dataset of lineage-traced VSMC-derived plaque cells from ApoE^−/−^ mice expressing the Confetti reporter induced by a *Myh11*-driven recombinase (*Myh11-CreERt2/Confetti/ApoE^−/−^*)^16^ (see Supplementary Material online, *Figure S7*). Contractile VSMC markers (*Myh11*, *Acta2*, *Cnn1*, and *Tagln)* were expressed in most clusters, but reduced in Cluster 6, Cluster 9 (which also expressed the chondrocytic markers chondroadheren (*Chad*) and *Sox9),* and Cluster 11 (which also expressed *Cd68*) (see Supplementary Material online, *Figure S7)*. *Tnfrsf11b, Fmod*, *Sfrp4*, *Lum*, *Dcn*, *Fn1*, *Col1a1*, and *Col3a1* were also mostly expressed in Clusters 6 and 9, and *Cdkn2a/p16* in Clusters 6, 8, 9, and 11 (see Supplementary Material online, *Figure S7*). *Tmem178b* was not detected. Expression of multiple de-differentiation marker genes with *Cdkn2a* in Clusters 6 and 9 may therefore represent VSMCs with a de-differentiated phenotype, and some of which are senescent VSMCs. This analysis suggests that several senescence and ‘fibromyocyte’ associated genes are expressed together in human and mouse atherosclerotic plaques.

### 3.6 Senescence promotes de-differentiated/’fibromyocyte’ VSMCs in atherosclerosis

**Figure 4:**
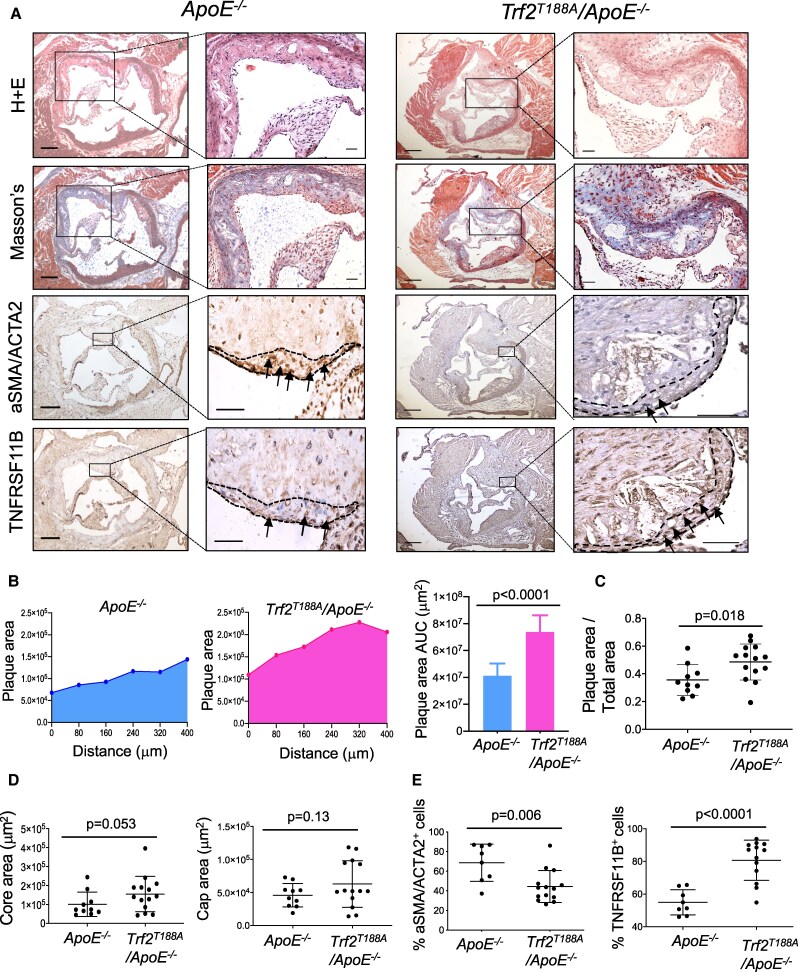
VSMC senescence promotes atherosclerosis and reduces ACTA2 + cells in the fibrous cap. (*A*) Aortic root atherosclerosis in *Myh11-Cre^ERt^*^2^*/Confetti/ApoE^−/−^ (ApoE^−/−^*) or *Myh11-Cre^ERt2^/Confetti/Sm22a-Trf2^T188A^*/*ApoE^−/−^ (Trf2^T188A^/ApoE^−/−^*) mice fed a high fat diet from 8-22w stained with H + E or Masson’s, or immunohistochemistry for aSMA/ACTA2 or TNFRSF11B. Dashed lines in insets indicate fibrous caps, and arrows indicate positive cells. Scale bar = 200 µm in lower and 50 µm in high power views. (*B–C*) Quantification of plaque area under the curve (AUC) at increasing distance across the aortic valve (*B*) and aortic root plaque area/total aortic root area (*C*). (*D*) Necrotic core or fibrous cap area in *ApoE^−/−^ or Trf2^T188A^/ApoE^−/−^* mice*. (E*) % aSMA/ACTA2-positive or TNFRSF11B-positive cells in fibrous caps in *ApoE^−/−^ or Trf2^T188A^/ApoE^−/−^* mice (*n* = 8–14, unpaired *t*-test).

To directly examine the effects of VSMC senescence on VSMC phenotypes *in vivo*, we used a mouse model of accelerated VSMC senescence due to telomere damage. *Sm22a-Trf2^T188A^* mice express a TRF2 point mutant driven by the arterial VSMC-specific minimal *Sm22a* promoter whose activity is maintained during phenotypic switching,^34^ and, unlike the full-length *Sm22a* promoter, is not expressed in bone marrow, peripheral blood cells or spleen.^4^ Cultured *Sm22a-Trf2^T188A^* VSMCs show normal proliferation initially but undergo progressive telomere damage during replication and cells undergo premature senescence.^3,10^*Sm22a-Trf2^T188A^/ApoE^−/−^* were crossed with *Myh11-Cre^ERt^*^2^*/Confetti/ApoE^−/−^* mice to generate *Myh11-Cre^ERt^*^2^*/Confetti/Sm22a-Trf2^T188A^*/*ApoE^−/−^ (Trf2^T188A^/ApoE^−/−^*) or *Myh11-Cre^ERt2^/Confetti/ApoE^−/−^ (ApoE^−/−^*) littermate control mice, administered tamoxifen to label VSMCs, and fed high fat diet for 14w. Blood pressure and serum lipids were similar between *Trf2^T188A^/ApoE^−/−^* and *ApoE^−/−^* mice, but serum IL1B and CXCL1 were increased or borderline increased (*P* = 0.05), respectively, in *Trf2^T188A^/ApoE^−/−^* mice (see Supplementary Material online, *Table S5*). Absolute and relative aortic root atherosclerosis areas and necrotic core and cap areas were analysed on serial sections across the valve (*Figure 4A-4D*). Atherosclerosis areas were significantly increased in *Trf2^T188A^/ApoE^−/−^* vs. *ApoE^−/−^* mice, with no overall change in core or cap areas (*Figure 4A–D*). However, despite similar numbers of Confetti-positive cells in the fibrous cap (see Supplementary Material online, *Figure S8*), aSMA/ACTA2-positive cells were reduced and TNFRSF11B-positive cells increased in caps in *Trf2^T188A^/ApoE^−/−^* vs. *ApoE^−/−^* mice. This analysis suggests that although *Trf2^T188A^/ApoE^−/−^* mice show similar VSMC numbers in fibrous caps, these cells have reduced contractile and increased fibromyocyte marker protein expression.

**Figure 5:**
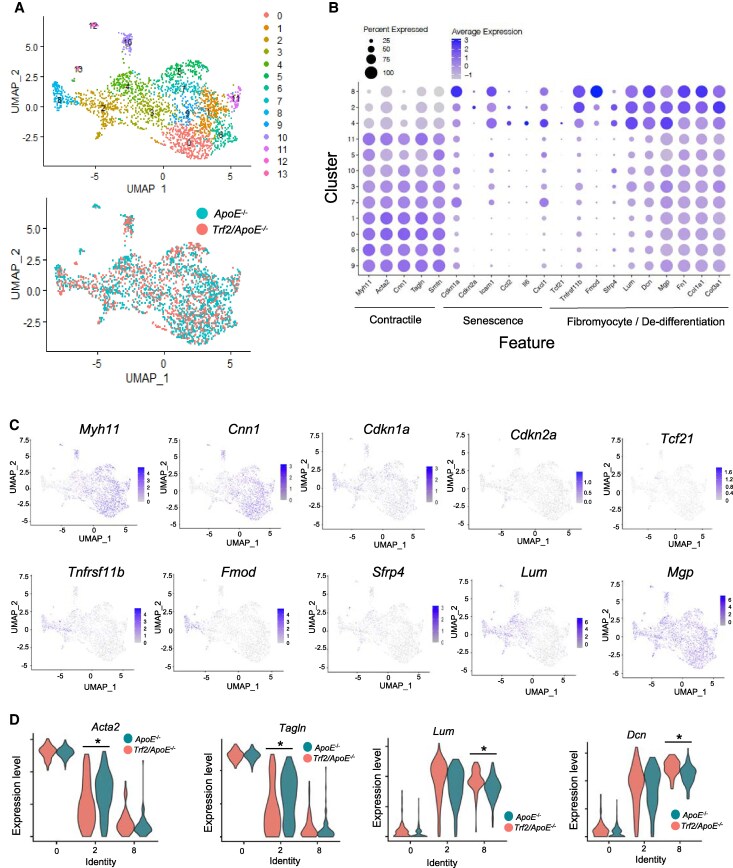
Expression of lineage and de-differentiation markers in mouse atherosclerosis. (*A*) UMAP of VSMCs isolated from atherosclerotic plaques of high fat-fed control *Myh11-CreERt2/Confetti/ApoE^−/−^ (ApoE^−/−^*) or *Myh11-CreERt2/Confetti/Sm22a-Trf2^T188A^*/*ApoE^−/−^ (Trf2^T188A^/ApoE^−/−^*) mice showing clustering (top panel) or labelled by genotype (lower panel). (*B*) Dot plot of selected contractile *(Myh11/Cnn),* senescence (*Cdkn1a/*C*dkn2a*) and fibromyocyte/de-differentiation markers *(Tcf21/Tnfrsf11b/Fmod/Srfrp4/Lum/Mgp).* Dot size represents the fraction of cells in each cluster that express the gene. Shade of colour represents scaled expression levels. (*C*) UMAP showing expression levels of selected genes associated with VSMC subsets, senescence- or fibromyocyte/de-differentiation genes on a log-transformed scale. (*D*) Violin plot of selected contractile (*Acta2, Tagln*) or de-differentiation marker (*Lum, Dcn*) gene expression in Clusters 0, 2, and 8 for *Trf2^T188A^/ApoE^−/−^* (green) and control *ApoE^−/−^* mice (orange). *adjusted *P* < 0.05, Wilcoxon Rank Sum test, |log_2_ FC|>0.25.

We generated scRNA-seq data from dissected plaques and identified 14 clusters of VSMC-derived cells after subsetting Confetti^+^ cells (929 cells from *Trf2^T188A^/ApoE^−/−^* and 1502 from control animals) (*Figure 5A*, Supplementary Material online, *Table S6* and Supplementary material online, *Figure S9*). Clusters 12 and 13 had <25 cells and were not considered further. Typical contractile VSMC markers (*Myh11, Cnn1, Acta2,* and *Tagln*) were detected at similarly high levels in Clusters 0, 1, 6, 9, and 11 (*Figure 5B* and *C*, Supplementary Material online, *Table S7*). In contrast, Clusters 2 and 8 showed lower contractile marker expression and higher expression of de-differentiation/fibromyocyte markers (*Tnfrsf11b, Fmod, Sfrp4, Lum, Dcn, Fn1, Col1a1,* and *Col3a1*) and *Cdkn2a,* suggesting that these clusters contain VSMCs with a de-differentiated phenotype and senescent VSMCs (*Figure 5B* and *C*, Supplementary Material online, *Table S7*). We assessed co-expression between fibromyocyte and senescence marker genes using Spearman’s rank correlation. There was significant positive correlation (FDR > 0.05) in expression of 52 of 60 possible gene-gene combinations, suggesting that senescence and fibromyocyte markers are also co-expressed by the same cells. Specifically, correlation analysis confirmed that *p16/Cdkn2a* expression correlates with *Tnfrsf11b, Fmod, a*nd *Sfrp4* (see Supplementary Material online, *Figure S10A*). Differential gene expression analysis between *Trf2^T188A^/ApoE^−/−^* and *ApoE^−/−^* VSMCs showed reduced *Acta2* and *Tagln* expression in *Trf2^T188A^/ApoE^−/−^* VSMCs in Cluster 2, and increased *Lum* and *Dcn* in *Trf2^T188A^/ApoE^−/−^* VSMCs in Cluster 8 (*Figure 5D*, Supplementary Material online, *Table S7*). GO analysis of genes with reduced expression in *Trf2^T188A^/ApoE^−/−^ vs. ApoE^−/−^* VSMCs showed enrichment for actin-filament-based processes in Cluster 2 and muscle contraction or development in both Clusters 2 and 8, respectively, whereas tissue migration and regulation of inflammatory responses were enriched for genes with increased expression in *Trf2^T188A^/ApoE^−/−^* compared to control mice in Cluster 8 (see Supplementary Material online, *Figure S10B,*Supplementary material online, *Table S8*). Overall, these data indicate that senescence reduces VSMC expression of contractile markers and increases expression of de-differentiation/fibromyocyte markers *in vivo* in atherosclerosis.

### 3.7 Senescence promotes a de-differentiated/’fibromyocyte’ VSMC state after artery ligation

In addition to senescence, VSMCs in atherosclerosis are continually exposed to inflammation and mitogens, stimuli that promote de-differentiation; it is therefore unclear whether senescence enhances de-differentiation, reduces re-differentiation or both. We therefore used carotid artery ligation, an established and less complex model of transient VSMC phenotypic switching that exhibits re-expression of contractile genes 28 days after surgery,^16,35^ when *Trf2^T188A^* mouse lesions also show increased senescence markers.^10^*Trf2^T188A^* mice showed increased neointima formation, but a smaller proportion of Confetti^+^ and aSMA/ACTA2 ^+^ Confetti^+^ cells compared to total neointimal cells, and higher proportion of aSMA/ACTA2^−^Confetti^−^ cells (see Supplementary Material online, Supplementary material online, *Figure S11A*), consistent with previous studies showing increased inflammatory infiltrate.^10^ Control and *Trf2^T188A^* mice also showed similar absolute numbers of neointimal Confetti^+^ cells, but *Trf2^T188A^* mice showed fewer Confetti^+^ cells expressing aSMA/ACTA2 or MYH11, and more expressing TNFRSF11B (see Supplementary Material online, Supplementary material online, *Figure S11B*–*D*). This analysis suggests that although neointimal VSMC numbers are similar, *Trf2^T188A^* VSMCs show reduced contractile and increased fibromyocyte marker protein expression.

**Figure 6:**
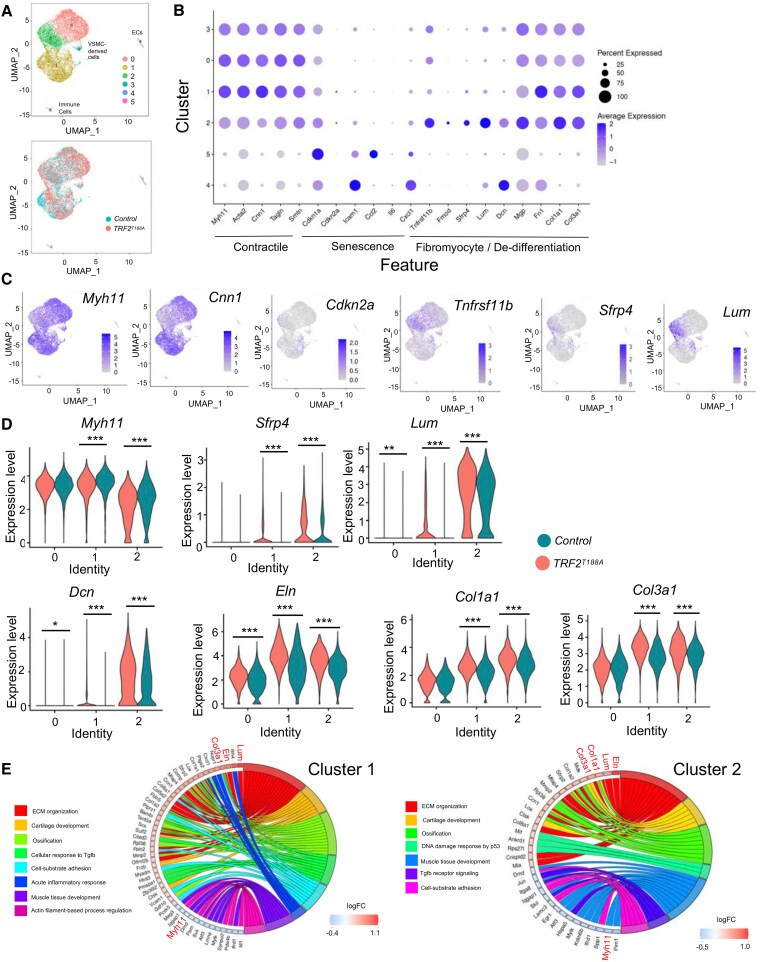
Expression of lineage and de-differentiation markers after carotid artery ligation. (*A*) UMAP of carotid artery cells isolated 28 days post ligation of control or *Trf2^T188A^* mice showing clustering (top) or labelled by genotype (lower panel). (*B*) Dot plot of lineage and de-differentiation markers. Dot size represents fraction of cells in each cluster that express the gene. Shade of blue represents scaled expression levels. (*C*) UMAP showing expression levels of genes associated with VSMC subsets, including selected contractile (*Myh11, Cnn1*), senescence− (*Cdkn2a*) or fibromyocyte/de-differentiation (*Tnfrsf11b, Sfrp4, Lum)* genes on a log-transformed scale. (*D*) Violin plot of selected contractile (*Myh11*) or fibromyocyte/de-differentiation marker (*Sfrp4/Lum/Dcn/Eln/Col1a1/Col3a1*) gene expression in Clusters 0–3 for *Trf2^T188A^* (green) and control mice (orange). Adjusted *P < 0.05, **P < 0.01, ***P < 0.001, Wilcoxon Rank Sum test, |log_2_ FC|>0.25. (*E*) Chord plots for selected Gene Ontology terms that are enriched in up- or down-regulated genes after carotid artery ligation in *TRF2^T188A^ vs*. control mouse VSMCs in Clusters 1 and 2. *adjusted *P* < 0.05, Wilcoxon Rank Sum test, |log_2_ FC|>0.25.

ScRNA-seq analysis of cells from control (*n* = 4967) and *Trf2^T188A^* (n = 5392) mice formed six clusters with contributions from both genotypes, representing ECs (Cluster 4), immune cells (Cluster 5) and VSMC-derived cells (expressing *Myh11*, *Cnn1*, *Acta2*, and *Tagln*; Clusters 0–3) (*Figure 6A–C*, Supplementary Material Online, Supplementary material online, *Table S9*, Supplementary Material Online, Supplementary material online, *Figure S12*). *Cdkn2a* was detected predominantly in Clusters 1 and 2 (*Figure 6B* and *C*). Compared to Cluster 0, Cluster 1 cells had higher expression of some de-differentiation-associated genes [*Fn1*, *Col1a1*, *Col3a1* (*Figure 6B,C*, Supplementary Material Online, Supplementary material online, *Table S10*)], while Cluster 2 cells had reduced contractile marker expression compared to other VSMC clusters, but increased levels of de-differentiation markers [*Fmod*, *Sfrp4*, *Mgp, Tnfrsf11b*, *Lum, Dcn* (*Figure 6B,C*, Supplementary Material Online, Supplementary material online, *Table S10*)]. Correlation analysis for senescence and fibromyocyte marker genes showed a significant positive correlation in the expression of 49 of 54 possible gene-gene combinations, suggesting their co-expression. Specifically, this analysis confirmed the positive correlation of *p16/Cdkn2a* with *Fmod* and *Sfrp4* (see Supplementary Material Online, Supplementary material online, *Figure S13*). Differential gene expression analysis revealed increased expression of de-differentiation-associated genes *Lum*, *Eln, Dcn, Col1a1*, *Col3a1,* and *Sfrp4* and lower expression of *Myh11* in *Trf2^T188A^* vs. wild-type cells in both Clusters 1 and 2, and *Eln* expression was higher in Cluster 0 in *Trf2^T188A^* cells (*Figure 6D*, Supplementary Material Online, Supplementary material online, *Table S10*). GO enrichment analysis of genes showing differential expression in *Trf2^T188A^* mice suggested differences in ECM organization, cartilage development, ossification, and TGFb response/signalling in Cluster 1 and Cluster 2 cells and acute inflammatory response in Cluster 1 (*Figure 6E*, Supplementary Material Online, Supplementary material online, *Table S11*). Overall, these data indicate that senescence results in VSMCs with reduced contractile markers and increased de-differentiation/fibromyocyte markers after injury, and that this might be related to perturbed TGFb signalling.

### 3.8 Trf2^T188A^ VSMCs show reduced re-differentiation to a contractile phenotype

To understand the relative contribution from enhanced de-differentiation vs. reduced re-differentiation, we examined contractile marker expression in uninjured aortas and cultured VSMCs of *Sm22a-Trf2^T188A^* and wild-type mice, and response of cultured VSMCs to TGFb or rapamycin, potent inducers of re-differentiation in VSMCs in culture.

**Figure 7:**
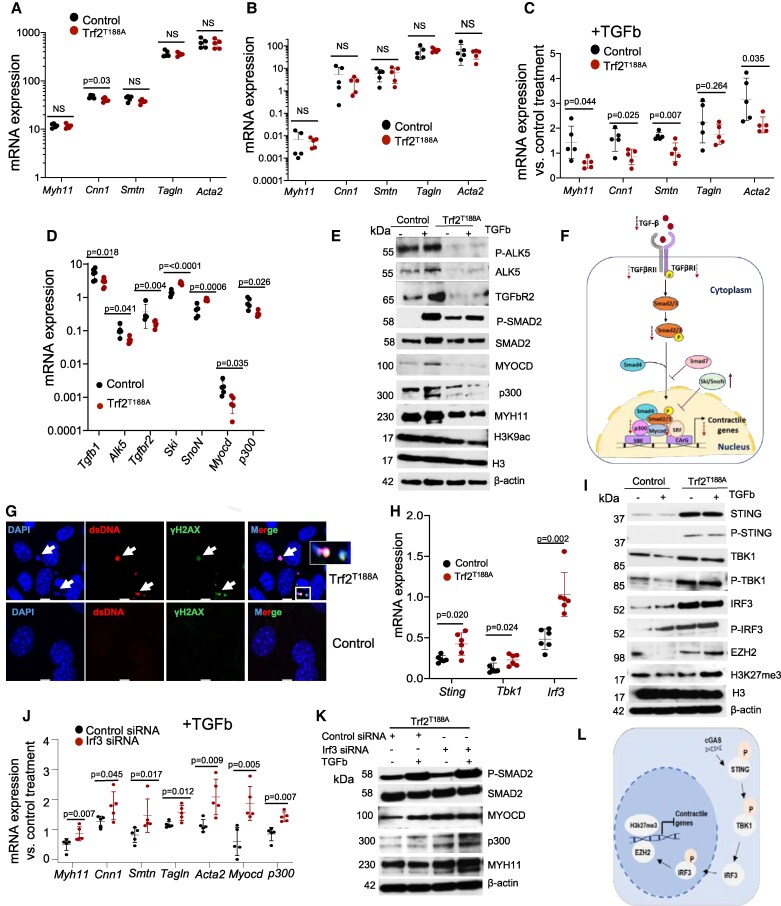
Senescence induces resistance to TGFb-mediated re-differentiation of VSMCs. (*A–B*) mRNA expression of VSMC contractile genes in aortas (*A*) or cultured VSMCs (*B*) derived from littermate control (wild-type) or *Trf2^T188A^* mice. (*C*) mRNA expression of contractile genes in cultured VSMCs from control or *Trf2^T188A^* mice treated with 10 ng/mL TGFb for 48 h vs. control treatment. (*D*) RT-QPCR for *Tgfb, Tgfbr1/Alk5, Tgfbr2*, and selected downstream regulators of Tgfb signalling. (*E*) Representative Western blot of Tgfb receptors, phospho-SMAD2, Myocardin, p300 and MYH11 in TGFb-treated VSMCs from control and *Trf2^T188A^* mice. (*F*) Schematic depicting changes in canonical Tgfb signalling in *Trf2^T188A^* VSMCs. (*G*) Immunocytochemistry of γH2AX^+^ cytosolic dsDNA (arrows) in control or *Trf2^T188A^* VSMCs. Scale bar = 5 µm. (*H*) RT-QPCR of STING pathway genes in VSMCs from control or *Trf2^T188A^* mice. (*I*) Representative Western blot of STING pathway components and H3K27me3 modification in control and *Trf2^T188A^* VSMCs. (*J*) RT-QPCR for VSMCs contractile markers, *Myocd* and *p300* in *Trf2^T188A^* VSMCs treated with *Irf3-targeting* or control siRNA followed by 10 ng/mL TGFb vs. control treatment. (*K*) Representative Western blot showing SMAD2 phosphorylation, MYOCD, p300 and MYH11 expression after treatment in (*J*). (*L*) Schematic illustrating components of cGAS/STING pathway downregulating VSMC contractile genes in *Trf2^T188A^* VSMCs. Data are means (SD) *n* = 5–7. Unpaired *t*-test.

*Myh11*, *Smtn*, *Tagln,* and *Acta2* transcripts were detected at similar levels in uninjured aortas and cultured VSMCs of *Trf2^T188A^* vs. control mice (*Figure 7A* and *B*). However, upregulation of *Myh11*, *Cnn1, Smtn* and *Acta2* after TGFb or rapamycin treatment was significantly blunted in *Trf2^T188A^* VSMCs vs. control cells (*Figure 7C*, Supplementary material online, *Figure S14A*), and upregulation of *Tnfrsf11b*, *Tmem178b* and *Sfrp4* were significantly enhanced by TGFb (see Supplementary material online, *Figure S14B*). These data suggest that whereas DNA damage in VSMCs does not significantly affect VSMC phenotype in intact aortas (where cells are not proliferating) or initial de-differentiation of contractile VSMCs in culture, the ability of de-differentiated VSMCs to re-express contractile markers is impaired.

We therefore examined receptor levels and expression of a range of intracellular regulators of Tgfb. Tgfb signalling occurs via both canonical and non-canonical pathways (see Supplementary Material Online, Supplementary material online, *Figure S15A*), the latter via activation/phosphorylation of p38, ERK1/2 and AKT kinases, but expression/phosphorylation of these kinases was similar in *Trf2^T188A^* vs. VSMCs (see Supplementary Material Online, Supplementary material online, *Figure S15B*). However, multiple components of the canonical (Smad) pathway were different between genotypes. For example, compared to control VSMCs, *Trf2^T188A^* had reduced levels of ligand (*Tgfb1),* transmembrane receptors (*Tgfbr2* and *Tgfbr1/Alk5,* that transduce Tgfb-mediated expression of contractile genes in human VSMCs^36^), and the transcriptional regulators myocardin (*Myocd*) and *p300*.^37,38^ In contrast, transcriptional co-repressors *Ski* and *SnoN*^39^ were significantly up-regulated in *Trf2^T188A^* vs. control VSMCs (*Figure 7D*). Western blots further showed that administered TGFb significantly up-regulated TGFBR2 and total and phosphorylated forms of TGFBR1/ALK5 in control but not *Trf2^T188A^* VSMCs, and similarly for SMAD2 phosphorylation, an early event following TGFb binding, MYOCD, and p300. In contrast, the active histone modification mark H3K9ac and MYH11 protein levels were down-regulated in *Trf2^T188A^* VSMCs (*Figure 7E*, Supplementary Material Online, Supplementary material online, *Figure S16A*). These results suggest that dysregulation of multiple components of canonical Tgfb signalling in *Trf2^T188A^* VSMCs could underlie their inability to re-express contractile markers after TGFb treatment (*Figure 7F*).

### 3.9 *Trf2^T188A^* VSMCs show cytoplasmic DNA and activation of STING/TBK1/IRF3

We have previously shown that human VSMCs expressing *Trf2^T188A^* show cytoplasmic DNA and activation of cGAS/STING/TBK1 intracellular DNA sensing pathways.^10^ cGAS/STING-induced activation of interferon-related factor (IRF3), the histone methyltransferase Enhancer Of Zeste 2 Polycomb Repressive Complex 2 Subunit (EZH2), and epigenetic modulation of VSMC contractile protein expression are all implicated in aortic aneurysm formation, which also shows DNA damage and VSMC phenotypic modulation.^40^ Activated IRF3 can also suppress Tgfb/SMAD signalling by preventing the association of SMAD proteins with Tgfb receptors and forming functional Smad transcriptional complexes.^41^

Cytoplasmic dsDNA expressing γH2AX was evident in *Trf2^T188A^* but not control VSMCs (*Figure 7G*), associated with upregulation of *Sting*, *Tbk1* and *Irf3* transcripts (*Figure 7H*). Both total and phosphorylated STING, TBK1, and IRF3 proteins were also up-regulated in *Trf2^T188A^* VSMCs cells and exogenous TGFb treatment did not alter their expression levels, suggesting pathway activation irrespective of TGFb treatment. In addition, TGFb treatment increased EZH2 and the repressive H3K27me3 epigenetic mark that it induces^42^ in *Trf2^T188A^* but not control VSMCs (*Figure 7I*, Supplementary Material Online, Supplementary material online, *Figure S16B*).

To examine the role of IRF3 in the impaired re-differentiation response to TGFb in *Trf2^T188A^* VSMCs, we transiently transfected *Trf2^T188A^* VSMCs with *Irf3* or control siRNA before TGFb administration. Silencing *Irf3* significantly increased TGFb-induced expression of contractile marker transcripts (Figure 7J), Smad2 phosphorylation and protein expression of MYOCD, p300 and MYH11 (*Figure 7K*, Supplementary Material Online, Supplementary material online, *Figure S17*). These results suggest that resistance of *Trf2^T188A^* VSMCs to TGFb-induced re-differentiation may be due to *Irf3*-induced repression of contractile proteins directly (*Figure 7L*) and/or TGFb receptors.

Finally, we examined whether cytoplasmic DNA without senescence could directly regulate the expression of VSMC contractile genes in response to Tgfb. Exogenous DNA transfection reduced TGFb-mediated upregulation of *Myh11*, *Smtn*, *Tagln*, *Acta2* without inducing senescence (*p16, Lamin b1)* or global DNA damage (p21, p53) markers in the whole population (see Supplementary Material Online, Supplementary material online, *Figure S18*). This suggests that cytoplasmic DNA, for example from DNA damage, can induce phenotypic switching even without senescence.

## 4. Discussion

We examined how DNA damage and senescence changes VSMC phenotype after replicative senescence (RS) of human VSMCs, doxorubicin treatment and recovery (D + R) in human and mouse VSMCs, and TRF2^T118A^ in mouse VSMCs *in vitro* and *in vivo*. Our important findings are as follows: (i) D + R and RS of human VSMCs induce many common genes, including de-differentiation/’fibromyocyte’ markers (*TNFRSF11B*, *FMOD)* and genes expressed by human fibroblast senescence *(TMEM178B, SFRP4*): (ii) D + R induces *Tmem178b, Sfrp4*, and *Tnfrsf11b* in mouse VSMCs; (iii) SFRP4, TNFRSF11B and FMOD are expressed in human atherosclerotic plaques predominantly by VSMCs; (iv) *SFRP4*, *TNFRSF11B*, *FMOD* and *CDKN2A* are expressed in scRNA-seq clusters representing de-differentiated and senescent VSMCs in human and mouse plaques *in vivo*; (v) *Sm22a-Trf2^T188A^* mice have increased atherosclerotic plaque size and neointimal areas after arterial injury, but similar VSMC numbers in fibrous caps or injury-induced neointimas compared to controls; however, VSMCs at these sites have reduced aSMA/ACTA2 and MYH11 and increased TNFRSF11B protein expression; (vi) *Trf2^T188A^* expression affects expression of multiple genes associated with Tgfb signalling, and reduces canonical TGFb signalling and the resulting induction of VSMC contractile markers after TGFb treatment; (vii) *Trf2^T188A^* VSMCs have cytosolic dsDNA and activation of the STING/TBK1/IRF3 pathway, while silencing IRF3 upregulates VSMC contractile gene expression after TGFb treatment; (viii) cytosolic dsDNA can downregulate contractile proteins even without senescence. Our data suggest that DNA damage and premature senescence cause VSMCs to maintain a de-differentiated phenotype, and that this may be partly due to impaired re-differentiation to a contractile phenotype.

### 4.1 Effect of cell senescence on VSMC phenotypic switching

DNA damage and senescent VSMCs accumulate during atherogenesis, and particularly in advanced lesions, most likely due to repeated rounds of replication, inflammation and cellular stress. VSMC de-differentiation is also seen in culture and during atherogenesis and early arterial injury, while partial re-differentiation can occur with the withdrawal of de-differentiation stimuli such as mitogens or oxidized lipids. It is therefore difficult to separate a direct effect of senescence on VSMC phenotypic switching from those due to common stimuli. However, our data shows that VSMC senescence has a direct effect on expression of contractile and de-differentiated VSMC markers and impairs re-differentiation, associated with activation and repression of specific intrinsic cell signalling pathways.

Senescence reduces VSMC proliferation and migration and induces inflammation, all of which may affect phenotypic switching.^3,4,10,11^ However, *Trf2^T188A^* did not reduce VSMC contractile marker expression in uninjured aortas or during de-differentiation *in vitro*, suggesting that *Trf2^T188A^*-induced DNA damage does not affect initial proliferation and de-differentiation of VSMCs. In contrast, *Trf2^T188A^* VSMCs showed reduced upregulation of many contractile markers *in vitro* after TGFb or rapamycin-induced re-differentiation. While confirmation of this finding *in vivo* will require detailed re-differentiation protocols and suppression of DNA damage signalling in senescent VSMCs, our data suggest that progressive DNA damage during replication may affect VSMC re-differentiation, causing persistence of de-differentiated VSMCs.

### 4.2 Fibromyocyte genes as senescence markers and potential targets in atherogenesis

We identified *TNFRSF11B*, *FMOD, TMEM178B* and *SFRP4* as ‘senescence-associated genes’, and significant correlation in the expression of many possible fibromyocyte and senescence gene-gene combinations in our scRNA-seq analysis; however, these 4 genes do not represent a specific VSMC ‘senescence signature’. Several studies have attempted to identify senescent cells based on gene expression data, including gene panels (e.g. SenMayo^42^ and Sencid^43^). However, panel performance has profound variation,^43^ indicating that senescent cell transcriptomes (e.g. in scRNA-seq datasets) are highly context-dependent, with considerable differences in expression signatures between cell types, disease state and severity. Furthermore, these 4 genes are up-regulated during VSMC phenotypic switching from multiple other stimuli and may have roles in atherogenesis and plaque stability that are not related to senescence. For example, in mice *Tnfrsf11b* can inhibit atherogenesis and promote fibrous cap formation,^26,27,28^ fibromodulin can promote LDL accumulation in plaques^29^ and *Sfrp4* reduces inflammation, oxidative stress, and plaque formation.^30,31^ In humans, both *SFRP4* and *FMOD* are related to ruptured plaques,^44^ but *FMOD* was reduced in ruptured vs. stable plaques while *SFRP4* was unchanged, and TNFRSF11B serum protein levels are associated with increased extent and progression of atherosclerosis and events (reviewed in^45^). *TMEM178B* is up-regulated in senescence of cultured human fibroblasts,^25^ but its function is unknown, although it may be a useful senescence marker in cultured human VSMCs.

### 4.3 DNA damage, cytoplasmic dsDNA and phenotypic modulation

Loss of lamin B1 in senescence results in cytoplasmic leakage of chromatin fragments, which is sensed by cGAS, resulting in activation of cGAS/STING/TBK1/IRF3 pathways.^46^ We find increased expression of *STING, TBK1* and *IRF3* transcripts and upregulation of their total and phosphorylated protein forms in *Trf2^T188A^* VSMCs, suggesting that DNA damage activates this pathway, and show that IRF3 suppresses Tgfb signalling and contractile gene expression in Trf2^T188A^ VSMCs. IRF3 shows structural similarity with SMAD proteins and interacts with inactive SMADs, inhibiting their activation/phosphorylation induced by Tgfb receptors.^41^ IRF3 also uses p300 as a transcriptional co-activator and competes for its use,^41^ supressing p300 and MYOCD availability and interacting with EZH2 to induce repressive epigenetic changes on contractile gene promoters. Although silencing IRF3 increases contractile gene expression in *Trf2^T188A^* VSMCs, manipulating IRF3 may not be useful therapeutically. IRF3 regulates the Type I interferon response that prevents/limits virus infections,^47^ and STING can also promote neointima formation via proliferation, migration, and phenotypic switching in VSMCs through NF-kB signalling.^48^

### 4.4 Limitations of our study

Dox1d + 21d and RS induced many similar genes. However, our studies do not identify whether VSMC DNA damage and senescence in atherosclerosis is due to RS or stressors such as oxidative stress or lipids, or both. Our findings also differ from other studies suggesting that senescent cells reduce VSMC migration through soluble growth factor inhibitors such as IGFBP3,^9^ while we find similar numbers of VSMCs in fibrous caps, and *IGFBP3* was not induced by senescent human VSMCs. Our atherosclerosis, ligation, and re-differentiation studies utilized *Sm22aTRF2^T188A^* mice and VSMCs, which may not completely recapitulate the phenotype of senescent VSMCs *in vivo*, and *TRF2^T188A^* might also be expressed in cells from other lineages that adopt a SMC-like phenotype *in vivo*, including endothelial cells (EndoMT) and bone marrow-derived cells.^49^ However, similar to *TRF2^T188A^* VSMCs, human plaque VSMCs show extensive telomere damage and premature senescence,^2^ and lineage tracing bone marrow-derived cells expressing specific SMC promoters showed only very rare cells in advanced lesions, and not in the fibrous cap.^49^ In addition, although we cannot exclude the possibility that *Sm22a-Trf2^T188A^* subtly affects SMC progenitor differentiation and arterial development, we find no difference in the expression of contractile or de-differentiation markers in adult vessels before fat feeding or injury. Finally, we find that *CDKN2A* expression (and by implication senescence) is low in human and mouse atherosclerosis and mouse carotid injury artery datasets compared with VSMC contractile and de-differentiated genes. However, scRNA-seq may underestimate cellular senescence, in part because senescent cells are under-represented due to their larger size, increased sensitivity to extraction conditions, are cleared by both killing and phagocytosis, and CDKN2A upregulation in senescence is mostly post-translational.

Translational perspective Risk factors for atherosclerosis such as smoking, diabetes and hyperlipidaemia induce DNA damage and senescence in vascular smooth muscle cells (VSMCs), both of which promote atherogenesis and features of unstable plaques. Senescence causes VSMCs to maintain a de-differentiated/fibromyocyte phenotype in atherosclerosis and after injury, associated with cytosolic DNA, dysregulation of multiple Tgfb signalling proteins, and resistance to re-differentiation. Preventing senescence may delay atherogenesis and promote plaque stability, in part by promoting re-differentiation to contractile VSMCs.

## 5. Conclusions

We find that senescence downregulates contractile genes and upregulates genes associated with a de-differentiated fibromyocyte phenotype in human and mouse VSMCs and maintains VSMCs expressing de-differentiated markers *in vivo*. Resistance to TGFb-mediated re-differentiation to a contractile phenotype due to cytosolic DNA-mediated activation of the cGAS/STING/TBK1/IRF3 pathway may partly explain how VSMC DNA damage reduces expression of contractile proteins in fibrous caps in atherosclerotic plaques, potentially promoting plaque instability and neointimal formation.

## Supplementary Material

cvaf102_Supplementary_Data

## Data Availability

The data underlying this article will be shared on reasonable request to the corresponding author. RNA-seq datasets (sc and bulk) have been deposited in GEO (GSE210406, GSE171663).
