# Supplementary material for: Premature cell senescence promotes vascular smooth muscle cell phenotypic modulation and resistance to re-differentiation
Source: Cardiovasc Res. 2025 Jun 10;121(9):1448–63. doi: 10.1093/cvr/cvaf102 (PMC12352304; doi:10.1093/cvr/cvaf102)
Supplement: cvaf102_Supplementary_Data [file cvaf102_supplementary_data.zip › Supplemental Methods, Tables and Figures.pdf]

Premature cell senescence promotes vascular smooth muscle cell phenotypic modulation and resistance to re-differentiation

Anuradha Kaistha, Sebnem Oc, Abel Martin Garrido, James CK Taylor, Maria Imaz, Matthew D Worssam, Anna Uryga, Mandy Grootaert, Kirsty Foote, Alison Finigan, Nichola Figg, Helle F Jørgensen, Martin Bennett

SUPPLEMENTARY MATERIAL

Table of contents

Supplementary Methods and References
Supplementary Tables S1-2 and 5
Supplementary Figures S1-17

Supplemental Methods
Isolation of human VSMCs
Aortas from patients undergoing cardiac transplant or aortic valve replacement were obtained under consent and used for isolation and culturing of VSMCs. After removal of the adventitia and endothelium, aortic tissue was cut into 2-3mm^2 pieces and placed in DMEM supplemented with 20% FCS, 100U/ml penicillin and 100ug/ml streptomycin. After 1month, cells were trypsinized and re-seeded in Smooth Muscle Cell Growth Medium 2 (Promocell, C22062,SMC-GM2) with supplements for subsequent passages. Media was replaced every 2-3 days. For doxorubicin experiments, passage 2 cells were treated with Doxorubicin (hydrochloride) (250nM, Cayman Chemicals) or vehicle (DMSO, Sigma-Aldrich) for 24h, washed three times with PBS and incubated with complete fresh media for 21d. For replicative senescence experiments, cells were considered senescent with no increase in cell number and minimal EdU incorporation over 14d, which occurred between passages 8-14, depending on individual cell cultures.

Isolation of mouse VSMCs
Mouse aortic VSMCs (mVSMCs) were isolated by enzymatic digestion. Briefly, the whole aorta was dissected from 8-12 wk old mice, cleaned of adventitial fat, and incubated for 10min at 37°C with 1 mg/mL Collagenase II (Gibco) and 1 Unit/mL Elastase (Worthington Biochemical) in DMEM (Sigma-Aldrich). The adventitia and endothelium were carefully removed and the cleaned vessels incubated with 2.5 mg/mL Collagenase II and 2.5 Units Elastase in DMEM at 37°C in 5% CO_2 to obtain a single cell suspension. Cells were centrifuged at 220g for 5 minutes at room temperature and the pellet re-suspended in DMEM/F12 medium (11320-074, Gibco). After 1 month, cells were switched to 10% FBS, split 1:2 when confluent and used in exponential growth (<passage 3). For experimental procedure, mVSMCs were treated with different concentrations of Doxorubicin or DMSO for 24h, washed 3X times with PBS and incubated in fresh complete media for 7d.

EdU incorporation
EdU assays were performed using Click-It Plus EdU Alexa 647 (C10340, Thermo Fisher Scientific) following the manufacturer's recommendations. Briefly, cells on glass coverslips were incubated with EdU (10uM) for 24 hours, fixed in 4% formaldehyde for 15 min and permeabilized in 0.5% Triton for 30 min. Cells were incubated with Alexa Fluor® picolyl azide 647 for 30 min to stain EdU$^+$ cells. Finally, samples were counterstained with DAPI to analyse total cell number and mounted on glass slides using Pro-Long Diamond Antifade Mountant (P36980, Invitrogen). Cells were analysed using Leica TCS SP5 confocal laser scanning microscope (Leica) at 20X and EdU$^+$ and total cells were quantified using Leica Advance Suite Advance Fluorescence Lite software.

SAβG activity
SAβG activity in vitro was assayed using the Senescence Cells Histochemical Staining kit (CS0030, Sigma-Aldrich) following the manufacturer's recommendations. Around 4x10^4 cells were seeded per well in a 12 well plate. 24h later cells were fixed in 1% fixtation buffer for 7 min. at RT followed by washing and incubation with staining mixture at 37^{0}C for 7-8 hrs. Cells were imaged using Nikon TMS-F microscope with GXCAMLITE camera. ImageJ (National Institutes of Health, Md, USA) software was used to quantify percentage of SAβG positive cells.

QPCR
mRNA from human and mouse VSMCs was isolated using NucleoSpin RNA columns (740955.5, Macherey-Nagel) and concentrations were determined by Nanodrop. cDNA was synthesized using an Omniscript RT kit (205111, Qiagen), and quantitative real-time PCR performed using Bio-rad SsoAdvanced™ Universal SYBR® Green Supermix (1725271) on a Bio-rad CFX-connect^{TM} cycler.

Expression Master Mix (Thermo Fisher Scientific) was used with a final concentration of 1X probe/primers for quantification of mRNA levels using Taqman. HMBS or RPL4 were used as "housekeeping" genes for mouse and GAPDH or RPL13A for human. All primers are listed in **Supplemental Table 1**.

Western blots
Cells were washed with ice cold PBS and lysed in RIPA lysis buffer supplemented with protease cocktail inhibitor I and II (Millipore) and deacetylase inhibitor Trichostatin A (T8552, Sigma) where required. Protein concentrations were calculated using BCA method (23227, Pierce BCA protein assay kit, Thermo Fisher scientific) using a BCA standard curve. Proteins were separated by SDS-PAGE, wet transferred to a 0.45 µm pore PVDF membrane and blocked for 1 h at room temperature in 5% milk in Tween20/TBS or 5% BSA in Tween20/TBS. Membranes were then incubated with primary antibodies overnight at 4°C. Next day

membranes were washed and incubated with a secondary-linked HRP antibody for 1h at room temperature and chemiluminescence detected using Amersham ECL detection kit (GE Healthcare). Primary/secondary antibodies used for Western blots are listed in **Supplemental Table 2**.

Confocal microscopy

Formalin-fixed, paraffin-embedded human carotid endarterectomy sections were fixed with 4% paraformaldehyde, permeabilized with 0.1% triton X-100 in PBS for 7-10 min and washed 3 times for 5 min with PBST (PBS+0.1% Tween 20) before blocking with 10% goat serum (X0907, DAKO) in Blocking buffer (1% BSA, 0.1% Tween 20) for 1h at room temperature (RT). Carotid sections were incubated with either primary antibodies: SFRP4 (1:500, ab154167, Abcam), Smooth muscle cell α-actin-Cy3 conjugated (1:1000, C6198, Sigma-Aldrich), CD31 (1:50, ab9498, Abcam), CD68 (1:100, 14-0689-82, Thermo Fisher Scientific), Fibromodulin (1:100, PA5-26250, Thermo Fisher scientific), TMEM178B (1:100, HPA048771, Atlas Antibodies), TNFRSF11b (1:100, PA5-26250, Thermo Fisher scientific) or rabbit (ab172730, Abcam) and mouse (ab18443,Abcam) IgG isotype controls diluted in 3% BSA for 1h at RT. TMEM178B staining was performed using Alexa Fluor 647 Tyramide SuperBoost kit (B40926, Thermo Fisher Scientific). For cytoplasmic DNA and γH2AX co-staining experiments, Control or Trf2^{T188A} VSMCs grown on coverslips were fixed with 4% paraformaldehyde, permeabilized with permeabilization buffer (0.1% Tween 20, 0.01% Triton-X in Phosphate Buffer Saline) for 5-7 min. to allow antibodies to cross the cellular but not nuclear membrane, washed 3 times for 5 min. with PBST, blocked for 1hr at room temperature in blocking buffer (1% BSA, 0.1% Tween 20, 2.25% Glycine) and then incubated with anti-dsDNA (1:1000, ab27156, Abcam), γH2AX (1:250,ab81299, Abcam) antibodies or control IgGs overnight in a humidified chamber at 4^0C. After incubation, sections and cells were washed 3 times for 5 min at room temperature followed by incubation with secondary antibodies: goat anti-rabbit Alexa Fluor 647 (1:500, ab150083, Abcam), goat anti-mouse Alexa Fluor 488 (1:500, A-11017, Invitrogen), goat anti-rabbit Alexa Fluor 488 (1:1000, A-11008, Invitrogen) or goat anti-mouse Alexa Fluor 594 (1:1000, A-11005, Invitrogen) for 1h at RT. Nuclei were stained with DAPI and slides were mounted in ProLong Gold for imaging.

Immunostaining of ligated carotid arteries

14 μm thick carotid artery sections were rinsed in PBS to remove OCT, permeabilized (0.5% Triton X-100 in PBS for 20 min), blocked for 1h at RT in blocking buffer (1% BSA/10% normal goat serum (Dako) in PBS) and incubated with primary antibodies: Smooth muscle cell a-actin (1:100), TNFRSF11b (1:100) and MYH11 (1:200) or isotype control at RT followed by goat anti-rabbit Alexa Fluor 647 secondary antibody. Nuclei were counterstained with DAPI and sections mounted in RapiClear 1.52 for confocal imaging.

Immunostaining and quantification of fibrous cap confetti cells

Formalin-fixed, paraffin-embedded aortic root sections from fat fed *Trf2^{T188A}/ApoE$^{-/-}$* vs. *ApoE$^{-/-}$* mice were permeabilized with 0.1% triton X-100 and blocked with 10% donkey serum (ab150129, Abcam) for 1h at room temperature. Sections were then incubated with antibodies against YFP (1:100, STJ140118, St John's lab), RFP (1:100, STJ140119, St John's lab), GFP (1:100, STJ140005, St John's lab) and mCerulean (1:100, STJ140282, St John's lab) for 1hr at room temperature. After washing 3x times sections were incubated with Alexa Fluor 488 conjugated secondary antibody (1:500, ab150129, Abcam) to stain components of Confetti. DAPI was used to stain the nuclei and sections were mounted in Prolong Gold. 40x images were captured using Leica TCS sp8 confocal microscope and confetti-positive cells in the cap region were counted using ImageJ software (NIH, MD, USA). Percent confetti positive cells were calculated by dividing positively stained cells by total number of DAPI$^+$ cells. The average of percentage in 2-3 fields was then taken as the final percentage of positive cells for each slide.

Imaging parameters

Images were captured using a Leica TCS SP8/SP5 laser scanning confocal microscope with a 63x oil immersion objective for human sections and 40x oil for mouse sections and cells, optical resolution of 1024x1024, with or without digital zoom. Images were captured under the same conditions as the isotype control during each confocal session to assure signal specificity. IMARIS 9.0.2 (BITPLANE AG, Switzerland) and LasX software were used for image processing, including brightness/contrast adjustment and generation of maximum projection intensities. Mouse carotid artery sections were analysed for expression of α-SMA,TNFRSF11B, MYH11 and confetti genes as described previously[1].

Blood pressure, serum lipids and cytokines

Blood pressures were determined by the tail cuff method using BP-2000 Blood Pressure analysis system (Visitech systems). Pre-measurements were taken before the actual measurements were recorded. Cytokine concentrations in mouse serum were measured using V-PLEX Mouse Proinflammatory Panel 1 following the manufacturer's recommendations. Serum lipids were analyzed using Siemens Dimension EXL analyzer, and high-density lipoproteins (HDL) analyzed using a Siemens Dimension RxL analyzer. Low-density lipoprotein (LDL) concentration was calculated from the triglyceride, HDL and cholesterol concentrations using the Friedwald formula (LDL= Cholesterol - HDL - (Triglycerides/2.2).

Histological Analysis
Mouse aortic roots were fixed overnight in 4% neutral buffered formalin, paraffin embedded and 5μm thick transverse serial sections cut 80μm apart from 0μm-400 μm. Atherosclerosis extent and composition were analyzed with Haematoxylin and Eosin (H&E) and Masson's Trichrome (HT15 kit, sigma Aldrich) staining. Low and high power images were captured using a bright-field microscope with Image-Pro Insight 9.1 (Media Cybernetics, MD, USA) imaging software. Crystalline clefts between collagen fibres were used to identify plaques, and fibrous caps were defined as the area rich in VSMCs (red) and proteoglycans (blue) on Masson's staining while necrotic cores were distinguished by their cholesterol-rich, matrix-poor and acellular content. The boundaries of lumen and outer wall were outlined and areas of fibrous cap (rich in SMC and extracellular matrix) and necrotic core (rich in cholesterol and cellular debris) were identified and quantified using ImageJ software.

For aSMA and TNFRSF11B immunohistochemical analysis, de-waxed and rehydrated sections were incubated in 120 mM sodium citrate buffer and endogenous peroxidase activity blocked with 3% hydrogen peroxide. After blocking in 10% BSA, sections were immunostained overnight for aSMA (1:400, BD Pharmingen 553322) and TNFRSF11B (1:2000). HRP-conjugated secondary antibody (anti-Rat 1:300, Vector BA4001) was applied the following day and sections visualized using DAB (DAB vector SK 4105). Percent positive aSMA and TNFRSF11B fibrous cap cells was calculated by dividing positively-stained cells by total number of cells using ImageJ software (NIH, MD, USA).

Data processing and analysis of RNAseq
Bulk RNA-seq counts were normalised and log-transformed using DESeq2 v.1.14.1[2] in R v.3.3.3 prior to clustering samples via principal component analysis (PCA) or hierarchical clustering. Differential expression analysis was performed as described previously[1] with all data available at Gene Expression Omnibus GSE171663. Enriched gene ontology (GO) terms were identified using g:Profiler[3] with an adjusted p-value threshold of 0.05. Selected GO terms were visualized along with the fold-change values for the associated differentially expressed gene through chord plots using GOplot v.1.0.2.

The human atherosclerosis dataset[4] was visualized using PlaqView 2.0 (https://plaqviewv2.pods.uvarc.io)[5] using author-provided clustering and annotation. Meta analysis of scRNA-seq data from different studies with human atherosclerotic and control arteries was obtained from https://zenodo.org/records/14007461[6]. The data was log-normalized and then subset to include only data from Wirka et al[4] (17,224 cells) using Seurat v.5.1.0[7,8] in R v.4.4.1. Correlation analysis was performed with the cells belonging to 'Pericytes (APOE+)', 'Pericytes (MYH11+)', 'Fibromyocytes', 'Myofibroblast', 'VSMC', 'Fibroblast', and 'Fibroblast (COL9A3+)' clusters, using the author-provided 'subclustering' metadata[6].

scRNA-seq data of plaques from *Sm22a-Trf2*^{T188A}*/ApoE*$^{-/-}$ and control mice (GSE210406) were analysed using Seurat v.4.3.0[7,9] in R v.3.6.2. Cells with <1,500 genes detected, >8% mitochondrial reads for control mice and <1,000 genes detected, >8% mitochondrial reads for *Sm22a-Trf2*^{T188A}*/ApoE*$^{-/-}$ mice were excluded from analysis. The datasets were log-normalised. Following principal component analysis (PCA) (18 PCs) and Louvain clustering (1.1 resolution) based on the top 2,000 highly variable genes (HVGs), the data was subsetted to cell clusters expressing Confetti markers, and HVG selection, PCA, and clustering were repeated (2,000 HVGs, 18 PCs, resolution 0.9). Cluster markers were identified with Wilcoxon rank sum test method, $\log_2$-fold change threshold of 0.5, adjusted p-value threshold of 0.05, and minimum fraction of detection in at least one of the conditions of 0.1 (*FindAllMarkers* function in Seurat). Differential expression testing between cells from *Trf2*^{T188A}*/ApoE*$^{-/-}$ and *ApoE*$^{-/-}$ mice was performed with Wilcoxon rank sum test method with the log2-fold change threshold of 0.25, and minimum fraction of detection in at least one of the conditions of 0.1 (*FindMarkers* function in Seurat) using FDR-adjusted p-values (p_{adj}<0.05)[10]. Significantly enriched GO terms were identified using clusterProfiler v.3.14.3 (*enrichGO* and *compareCluster* functions)[11] with org.Mm.eg.db v.3.10.0 and the Benjamini-Hochberg adjusted p-value threshold and q-value threshold

of 0.05. Chord plots for selected GO terms and the corresponding differentially expressed genes were generated using GOplot v.1.0.2[12].

Analysis of scRNA-seq profiles from *Sm22a-Trf2*^{T188A} or wild-type littermate control mice (GSE210406) was performed using Seurat v.4.3.0[7,9] in R v.4.2.2. Cells with <1,200 genes detected, >6% mitochondrial reads, or >40% reads from the most highly expressed genes were excluded from analysis. Following log-normalisation, PCA (17 PCs) and clustering (Louvain algorithm, resolution 0.2) was performed with the top 2,000 HVGs. Identification of cluster marker genes, differential expression analysis between cells from *Trf2*^{T188A}*/ApoE*$^{-/-}$ and *ApoE*$^{-/-}$ mice as well as GO term analyses and visualizations were performed as above.

Pairwise correlation analysis was performed within the contractility, senescence and fibromyocyte/de-differentiation associated genes by using scran package (PMID: 27909575), correlatePairs function, which is based on Spearman's rank correlation.

References

1. Uryga AK, Grootaert MOJ, Garrido AM, Oc S, Foote K, Chappell J, Finigan A, Rossiello F, d'Adda di Fagagna F, Aravani D, Jorgensen HF, Bennett MR. Telomere damage promotes vascular smooth muscle cell senescence and immune cell recruitment after vessel injury. *Commun Biol*. 2021;**4**:611.
2. Love MI, Huber W, Anders S. Moderated estimation of fold change and dispersion for RNA-seq data with DESeq2. *Genome Biol*. 2014;**15**:550.
3. Raudvere U, Kolberg L, Kuzmin I, Arak T, Adler P, Peterson H, Vilo J. g:Profiler: a web server for functional enrichment analysis and conversions of gene lists (2019 update). *Nucleic Acids Res*. 2019;**47**:W191-W198.
4. Wirka RC, Wagh D, Paik DT, Pjanic M, Nguyen T, Miller CL, Kundu R, Nagao M, Coller J, Koyano TK, Fong R, Woo YJ, Liu B, Montgomery SB, Wu JC, Zhu K, Chang R, Alamprese M, Tallquist MD, Kim JB, Quertermous T. Atheroprotective roles of smooth muscle cell phenotypic modulation and the TCF21 disease gene as revealed by single-cell analysis. *Nat Med*. 2019;**25**:1280-1289.
5. Ma WF, Turner AW, Gancayco C, Wong D, Song Y, Mosquera JV, Auguste G, Hodonsky CJ, Prabhakar A, Ekiz HA, van der Laan SW, Miller CL. PlaqView 2.0: A comprehensive web portal for cardiovascular single-cell genomics. *Front Cardiovasc Med*. 2022;**9**:969421.
6. Bleckwehl T, Babler A, Tebens M, Maryam S, Nyberg M, Bosteen M, Halder M, Shaw I, Fleig S, Pyke C, Hvid H, Voetmann LM, van Buul JD, Sluimer JC, Das V, Baumgart S, Kramann R, Hayat S. Encompassing view of spatial and single-cell RNA sequencing renews the role of the microvasculature in human atherosclerosis. *Nat Cardiovasc Res*. 2025;**4**:26-44.
7. Satija R, Farrell JA, Gennert D, Schier AF, Regev A. Spatial reconstruction of single-cell gene expression data. *Nat Biotechnol*. 2015;**33**:495-502.
8. Hao Y, Hao S, Andersen-Nissen E, Mauck WM, 3rd, Zheng S, Butler A, Lee MJ, Wilk AJ, Darby C, Zager M, Hoffman P, Stoeckius M, Papalexi E, Mimitou EP, Jain J, Srivastava A, Stuart T, Fleming LM, Yeung B, Rogers AJ, McElrath JM, Blish CA, Gottardo R, Smibert P, Satija R. Integrated analysis of multimodal single-cell data. *Cell*. 2021;**184**:3573-3587 e3529.
9. Stuart T, Butler A, Hoffman P, Hafemeister C, Papalexi E, Mauck WM, 3rd, Hao Y, Stoeckius M, Smibert P, Satija R. Comprehensive Integration of Single-Cell Data. *Cell*. 2019;**177**:1888-1902 e1821.
10. Benjamini Y, Drai D, Elmer G, Kafkafi N, Golani I. Controlling the false discovery rate in behavior genetics research. *Behav Brain Res*. 2001;**125**:279-284.
11. Yu G, Wang LG, Han Y, He QY. clusterProfiler: an R package for comparing biological themes among gene clusters. *OMICS*. 2012;**16**:284-287.
12. Walter W, Sanchez-Cabo F, Ricote M. GOplot: an R package for visually combining expression data with functional analysis. *Bioinformatics*. 2015;**31**:2912-2914.

Supplemental Table S1
Primer sequences used in QPCR or Taqman.

Human primers

Gene	Forward 5' to 3'	Reverse 5' to 3'
SFRP4	ACGAGCTGCCTGTCTATGAC	TGTCTGGTGTGATGTCTATCCAC
TMEM178B	ATGAGTGGCATGCCCTACAC	ACGTACTGCATAAGGCCTCG
FMOD	AGTCAACACCAACCTGGAGAA	CACCTGCAGCTTGGAGAAGTT
TNFRSF11B	GAATGCAAGGAAGGGCGCTA	TATTTCGCTCTGGGGTTCCAGC
CDKN2A	CCAACGCACCGAATAGTTACG	GCGCTGCCCATCATCATG
RPL13A	CGAGGTTGGCTGGAAGTACC	CCGTAGCCTCATGAGCTGTT
ACTA2	AGACCCTGTTCCAGCCATC	TGCTAGGGCCGTGATCTC
**Taqman probes**		
p21	QT00062090	Qiagen
LmnB1	Hs01059210_m1	Thermo Fisher Scientific
GAPDH	Hs02786624_g1	Thermo Fisher Scientific

Mouse primers

Gene	Forward 5' to 3'	Reverse 5' to 3'
CDKN2A	TTGAGCAGAAGAGCTGCTACGT	CGTACCCCGATTCAGGTGAT
p21	GCAGATCCACAGCGATATCC	CAACTGCTCACTGTCCACGG
LMNB1	GGGAAGTTTATTCGCTTGAAGA	ATCTCCCAGCCTCCCATT
p53	ACCGCCGACCTATCCTTACC	TCTTCTGTACGGCGGTCTCTC
IL6	CTCTGCAAGAGACTTCCATCCA	AGTCTCCTCTCCGGACTTGT
SFRP4	AGGCAATAGTCACTGACCTTCC	CCTTTTTGCACTTGCACCGAT
TMEM178B	ACCATTCGCCAGGATGAGTG	ACGTACTGCATAAGGCCTCG
FMOD	AGCAGTCCACCTACTACGACC	CAGTCGCATTCTTGGGGACA
TNFRSF11B	ACCCAGAAACTGGTCATCAGC	CTGCAATACACACACTCATCACT
TCF-21	AGGTCATTCTCTGGTTTGCC	GCTACATCGCTCACTTAAGGC
Decorin (DCN)	TGCGATCCCTCAAGGTCTGCCT	TTGGCCAGACTGCCATTCTCCA
Lumican (LUM)	TCGAGCTTGATCTCTCCTAT	TGGTCCCAGGATCTTACAGAA
Matrix Gla Protein	AAGAGAGTCCAGGAACGCAA	GGTTGTAGGCAGCGTTGTAG
MYH11	CTGAGGGAGCGATACTTCTC	TGTAGCATGCTTCTGTAGGC
TAGLN	AGTATGACGAGGAGCTGGAG	AAGAATTGAGCCACCTGTTC
ACTA2	AGATCTGGCACCACTCTTTC	GTGAGTCACACCATCTCCAG
CNN1	TCATCAAAGCCATTACCAAG	TGCCTTCTCTCAACTTCTCC
SMTN	GCAAGGCCATGATTGAGAAG	CAGCAACATCTGCTTGATGC
TGFβ1	GTGGAAATCAACGGGATCAGC	GTTGGTATCCAGGGCTCTCC
ALK-5	TGCTCCAAACCACAGAGTAGGC	CCCAGAACACTAAGCCCATTGC
TGFβRII	CTACCACGGCTTCACTCTGG	GCTGGTGGTGTATTCTTCCGA
MYCOD	CCAACACCTTGCCCAGTTATC	GGAGCTTGTGCTGCCAAAG
p300	GACTCCACAGCCTGGTTTGA	GCGAGGTCCATAGCCCATAG
SKI	GTTCCTCCAATAAGAGCCTCGG	CTCGAATCAAAGCTGGGAGGT
SnoN	CGGCGGGCACAGATCAATTA	AAATGGTGATCTGCTGCTCAGT
STING	AAGTCTCTGCAGTCTGTGAAG	TGTAGCTGATTGAACATTCGGA
TBK1	GACATGCCTCTCTCCTGTAGTC	GGTGAAGCACATCACTGGTCTC
IRF3	CGGAAAGAAGTGTTGCGGTTAGC	CAGGCTGCTTTTGCCATTGGTG
RPL4	CGCAACATCCCTGGTATTACT	ACTTCCGGAAAGCACTCTCCG
HMBS	ACTGGTGGAGTATGGAGTCTCAGATGG C	GCCAGGCTGATGCCCAGGTT

Supplemental Table S2
Antibodies used for Western blots

Antibody	Catalog number	Company	Species	Dilution
**Primary**				
p16Ink4a	10883-1-AP	ProteinTech	Rabbit	1/1000
p21 (12D1)	2947	Cell signaling	Rabbit	1/1000
p53 (DO-7)	48818	Cell signaling	Mouse	1/1000
GAPDH (14C10)	2118	Cell signaling	Rabbit	1/1000
LmnB1 (M-20)	Sc-6217	Santa Cruz	Goat	1/250
SFRP4	ab154167	Abcam	Rabbit	1 2500
TMEM178B	HPA048771	Atlas antibodies	Rabbit	1/200
TNFRSF11B	PA5-86053	Invitrogen	Rabbit	1/1000
FMOD	PA5-26250	ThermoFisher scientific	Rabbit	1/1000
p38 MAPK	9212	Cell Signalling	Rabbit	1/1000
Phospho-p38 MAPK	28796-1-AP	Proteintech	Rabbit	1/1000
AKT	60203-2	Proteintech	Mouse	1/1000
Phospho-AKT	66444-1-1g	Proteintech	Mouse	1/1000
ERK1/2	11257-1-AP	Proteintech	Rabbit	1/1000
Phospho-ERK1/2	28733-1-AP	Proteintech	Rabbit	1/2000
TGFβRI/ALK-5	ab31013	Abcam	Rabbit	1/200
Phospho-ALK-5	STJ91351-50	St John's lab	Rabbit	1/100
TGFβRII	66636-1-1g	Proteintech	Mouse	1/1000
SMAD2	5339S	Cell Signaling	Rabbit	1/2000
Phospho-SMAD2	AB3849-1	Millipore	Rabbit	1/2000
p300	05-257	Millipore	Mouse	1/2000
Myocardin	MAB4028	R&D Systems	mouse	1/1000
MYH11	ab82541	Abcam	Rabbit	1/1000
STING	13647S	Cell Signaling	Rabbit	1/1000
Phospho-STING	D8F4W	Cell Signaling	Rabbit	1/1000
TBK1	3013S	Cell Signaling	Rabbit	1/800
Phospho-TBK1	5483S	Cell Signaling	Rabbit	1/1000
IRF3	4302S	Cell Signaling	Rabbit	1/1000
Pospho-IRF3	S396	Cell Signaling	Rabbit	1/1000
EZH2	21800-1-AP	Proteintech	Mouse	1/1000
H3K9ac	9649	Cell Signaling	Rabbit	1/1000
H3K27me3	39435	Activemotif	Mouse	1/1000
α/β tubulin	2148	Cell signalling	Rabbit	1/1000
β-Actin	A5-441	Sigma-Aldrich	Mouse	1/10 000
**Secondary**				
Anti Rabbit	7074	Cell signaling		1/2000
Anti Mouse	LNA931V/AG	Amersham		1/2000
Anti Goat	sc-2354	Santa Cruz		1/1000

Supplementary Table 5

Blood pressure (n=7-14), serum lipids and cytokines (n=11-19) of *ApoE*$^{-/-}$ and *TRF2*^{T188A} mice after 16 weeks of high fat diet. Data are means (SD). Unpaired Student t-test.

	ApoE$^{-/-}$	*Trf2*^{T188A}/*ApoE*$^{-/-}$	**Statistical analysis (p value)**
**Serum lipids (mmol/L, mean/SD)**			
Cholesterol	14.81 (3.54)	14.66 (3.76)	0.91
LDL	12.73 (3.55)	13.35 (3.38)	0.64
HDL	0.63 (0.25)	0.57 (0.18)	0.48
Triglycerides	3.19 (1.61)	2.40 (0.85)	0.15
**Serum cytokines (pg/ml, mean/SD)**			
IFN-γ	0.54 (0.09)	0.64 (0.21)	0.08
IL10	232.3 (106.00)	152.70 (64.10)	0.18
IL1β	0.5243 (0.44)	1.101 (0.86)	0.03
IL6	31.94 (21.2)	33.18 (19.00)	0.77
TNFα	36.42 (15.20)	32.42 (10.00)	0.32
IL5	11.78 (5.5)	11.93 (6.05)	0.94
IL2	1.629 (0.31)	1.929 (0.72)	0.15
CXCL1	57.15 (30.00)	104.6 (57.14)	0.05
**Blood Pressure (mmHg, mean/SD)**			
Systolic BP	104.60 (19.54)	111.30 (10.39)	0.3087
Diastolic BP	35.84 (21.66)	39.78 (17.75)	0.6602

Supplemental Figures

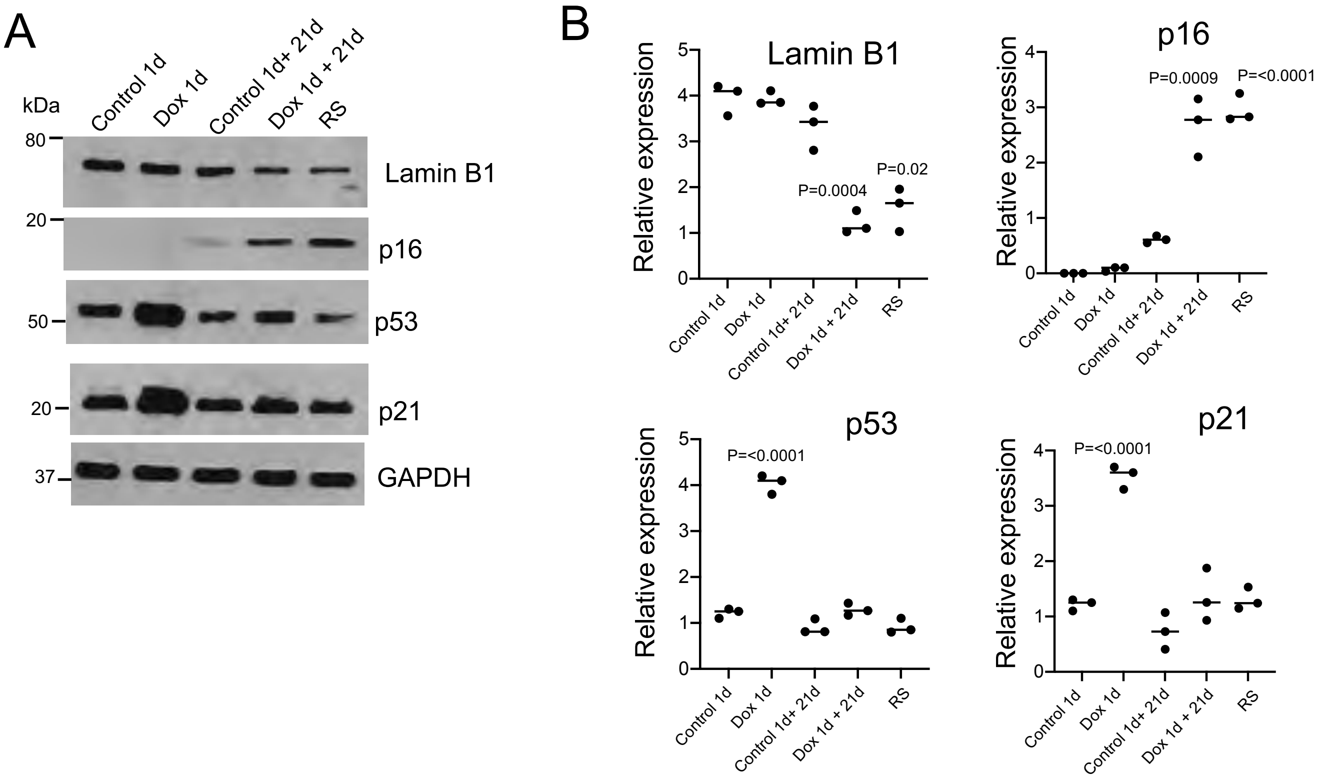

Figure S1 Senescence markers in human and mouse VSMCs
(A-B) Western blot **(A)** and relative expression **(B)** vs. GAPDH for Lamin B1, p16, p21, and p53 for proliferating human VSMCs (Control 1d), after 24h treatment with 250nM doxorubicin (Dox 1d), after an additional 21 days in culture in control conditions (control 1d + 21d) or after doxorubicin (Dox 1d+ 21d), or at replicative senescence (RS). n=3 human VSMC isolates, 1-way ANOVA**.**

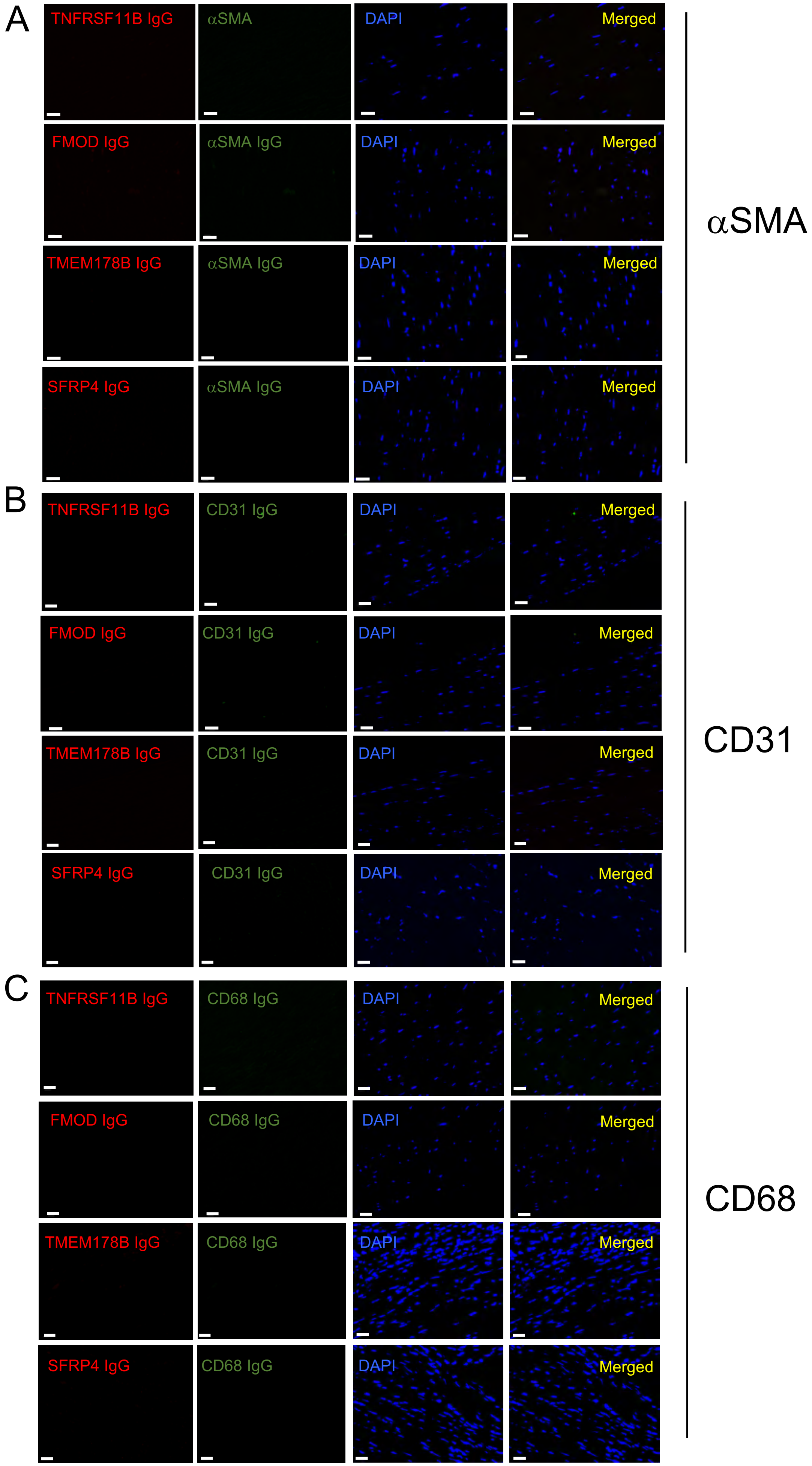

Figure S2, Confocal microscopy using Isotype control antibodies for TNFRSF11B, FMOD, TMEM178B, and SFRP4, together with αSMA/ACTA2 **(A)**, CD31 **(B)**, or CD68 **(C)** with DAPI. Scale bars = 10μm.

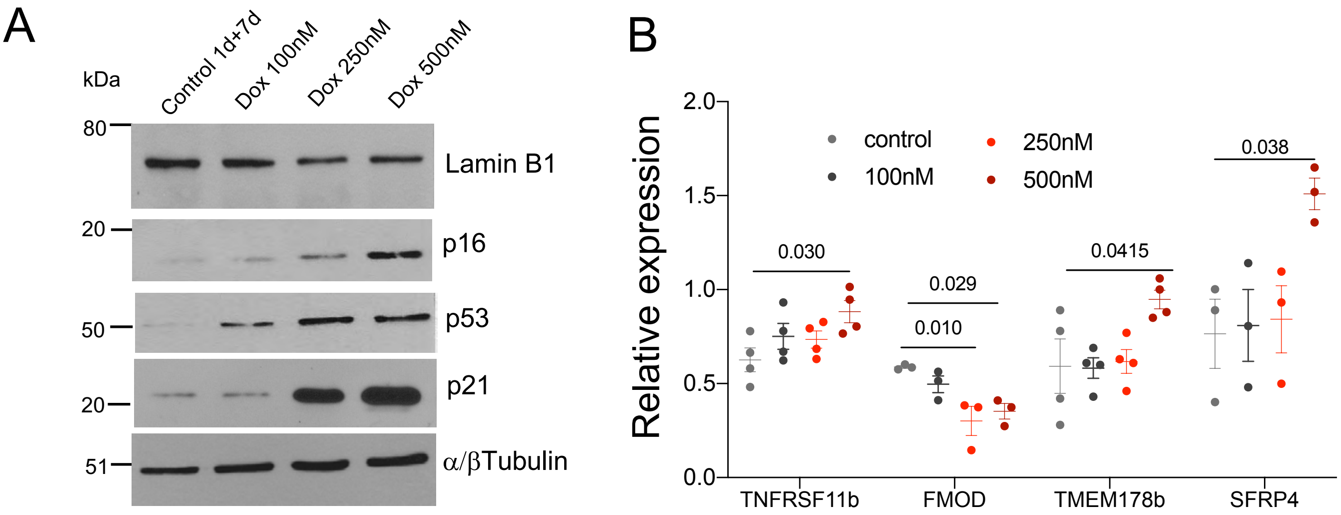

Figure S3
Western blot for Lamin B1, p16, p53 and p21 (n=2-5 mouse VSMC isolates) **(A)** or quantification for senescence-associated proteins **(B)** in mouse VSMCs treated with increasing concentration of Dox for 1d followed by 7d recovery. (n=3-4). 1-way ANOVA

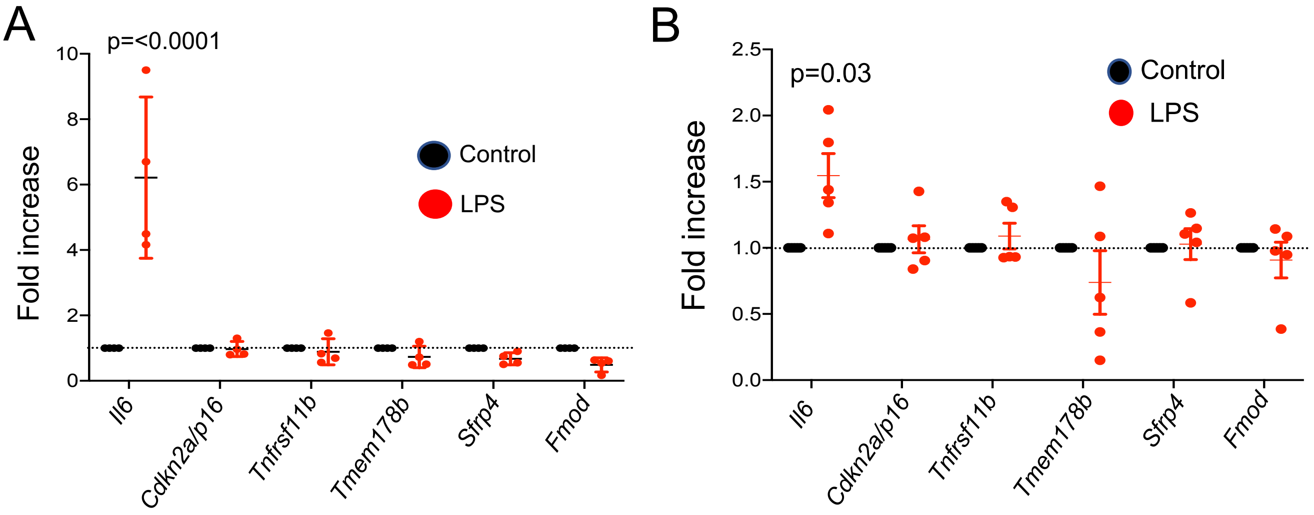

Figure S4
Fold increase in mRNA expression of Il6 or a range of senescence-associated (*Sfrp4, Tmem178b, cdkn2a/p16*) and/or 'fibromyocyte' genes (*Fmod, Tnfrsf11b*) in mouse VSMCs treated with LPS for 24h **(A)** or 24h treatment followed by 7d recovery **(B)** vs. vehicle control. Data are mean (SEM), n=4-5. Unpaired t test.

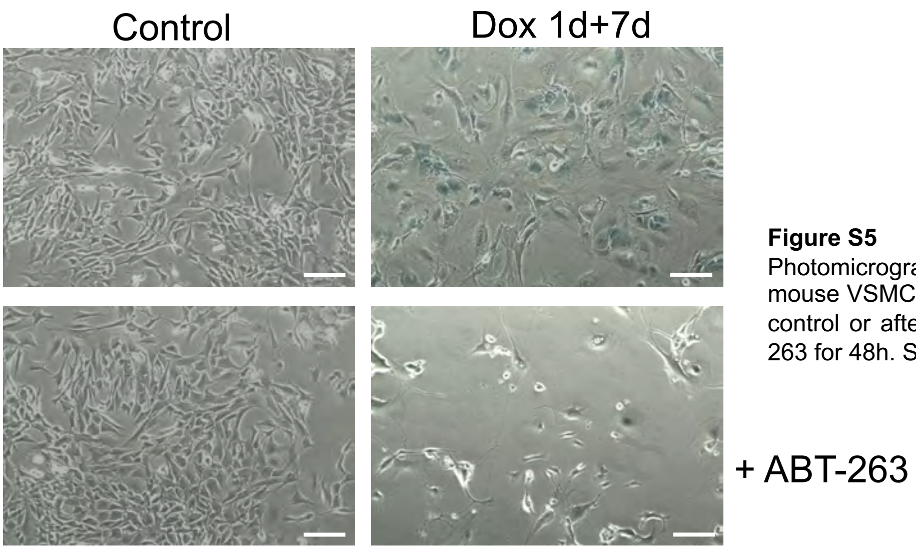

Figure S5
Photomicrograph of SAβG^{+} cells in mouse VSMCs treated with vehicle control or after dox 1d+7d ± ABT-263 for 48h. Scale bars = 50μm.

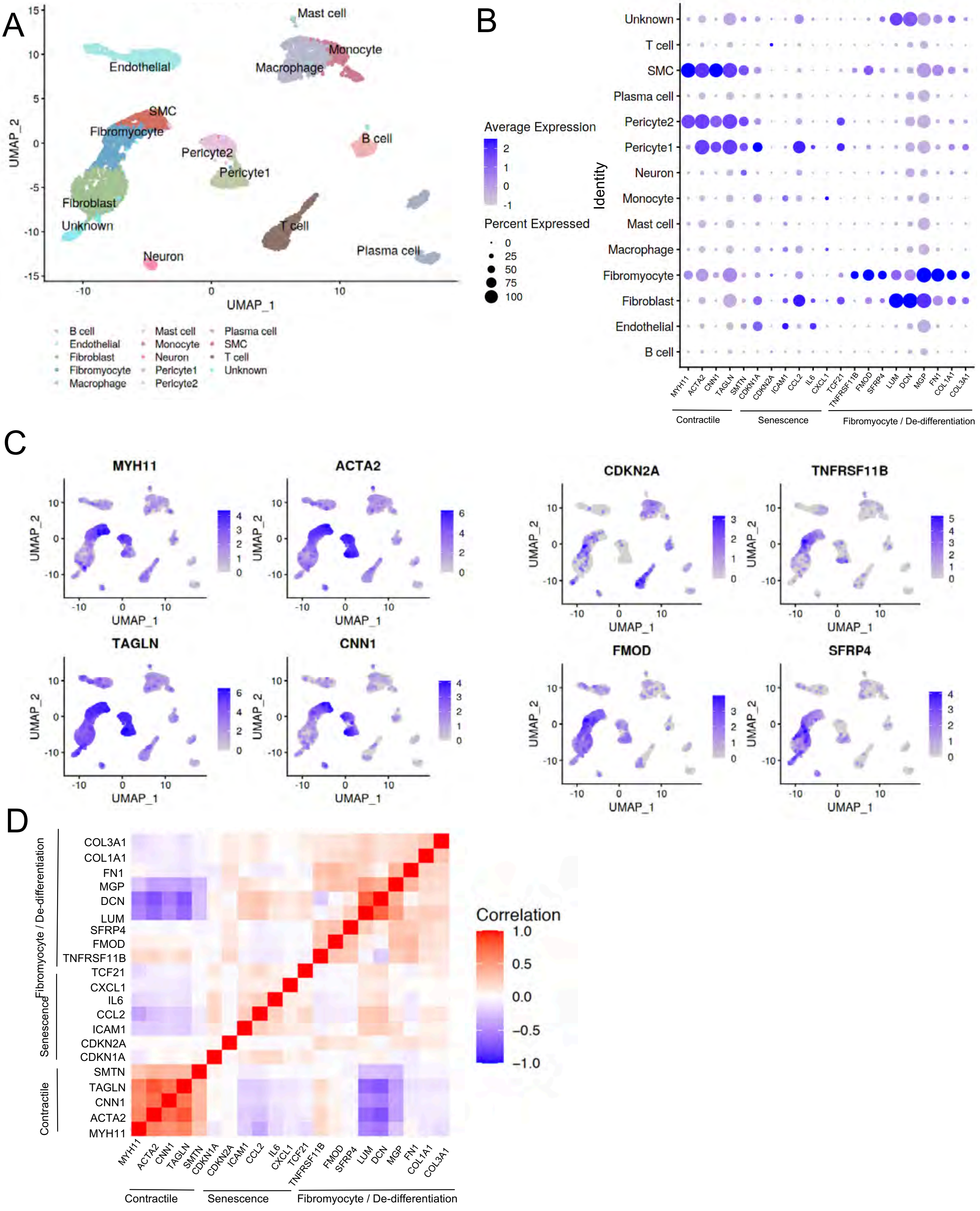

Figure S6 Expression of contractile, senescence and fibromyocyte / de-differentiation markers in human atherosclerotic plaques

(A) Uniform Manifold Approximation and Projection (UMAP) of scRNA-seq dataset from human coronary plaques (GSE131778) showing author-provided cell type annotation. (**B**) Expression dot plot showing expression of genes associated with VSMC contraction, senescence or de-differentiation in plaque cell clusters. Dot size represents fraction of cells in each cluster that express the gene. Shade of blue represents scaled expression levels. **(C)** UMAP showing expression levels of genes associated with VSMC subsets, including selected contractile (*MYH11, ACTA2, TAGLN, CNN1)*), senescence- (*Cdkn2a*) or fibromyocyte/de-differentiation (*TNFRSF11B, FMOD, SFRP4)* genes generated with Plaqview[5]. **(D)** Correlation heatmap of contractile, senescence and fibromyocyte / de-differentiation markers within VSMC, fibromyocyte, myofibroblast, fibroblast, and pericyte clusters

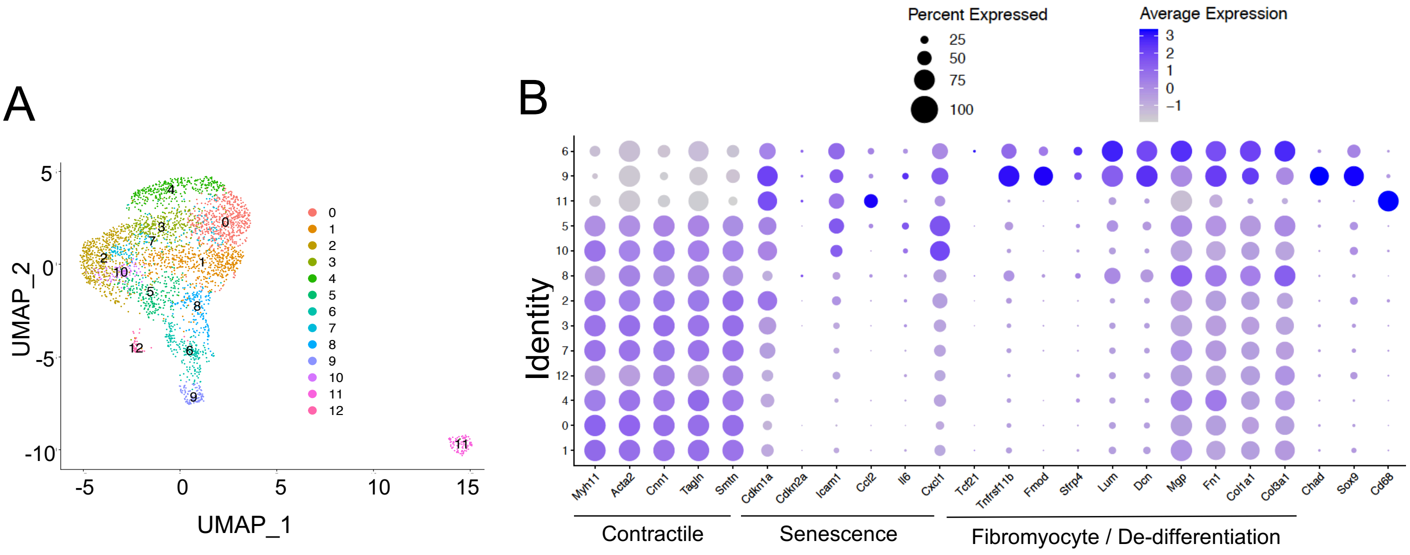

Figure S7 Expression of contractile, senescence and de-differentiation / fibromyocytic markers in mouse atherosclerotic plaques

(A) UMAP showing clusters of VSMC-derived (lineage-labelled) cells from high-fat diet-induced atherosclerotic plaques from *Myh11-Cre^{ERt2}/Confetti/ApoE$^{-/-}$* mice (GSE117963). (**B)** Dot plot showing expression of lineage markers. Dot size represents fraction of cells in each cluster that express the gene. Shade of blue represents scaled expression levels.

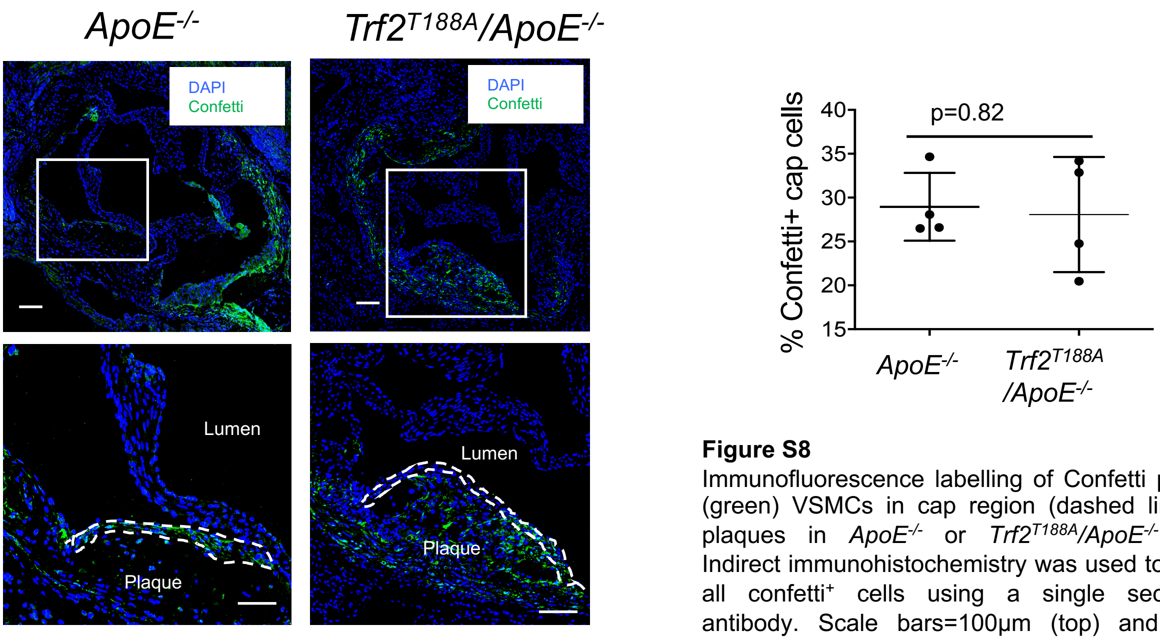

Figure S8

Immunofluorescence labelling of Confetti positive (green) VSMCs in cap region (dashed lines) of plaques in *ApoE$^{-/-}$* or *Trf2^{T188A}/ApoE$^{-/-}$* mice. Indirect immunohistochemistry was used to detect all confetti^{+} cells using a single secondary antibody. Scale bars=100µm (top) and 50µm (bottom). n=4. Unpaired t test.

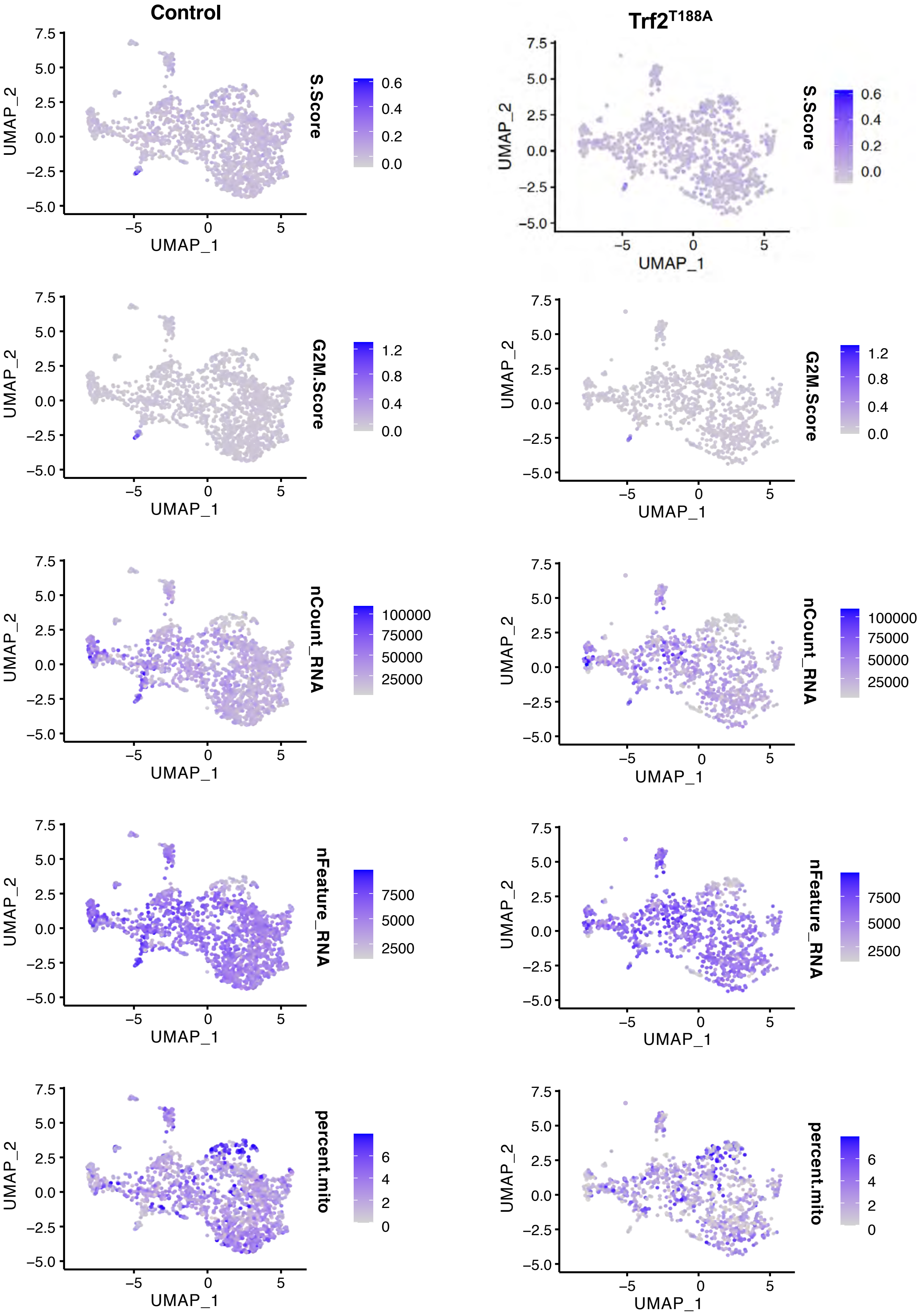

Figure S9
UMAPs showing S-phase score, G2-M phase score, number of unique molecular Identifiers (UMIs, nCount_RNA), number of genes (nFeatures), and percent mitochondrial genes for VSMCs isolated after high fat diet from control Apoe$^{-/-}$ or Trf2^{T188A}/Apoe$^{-/-}$ mice.

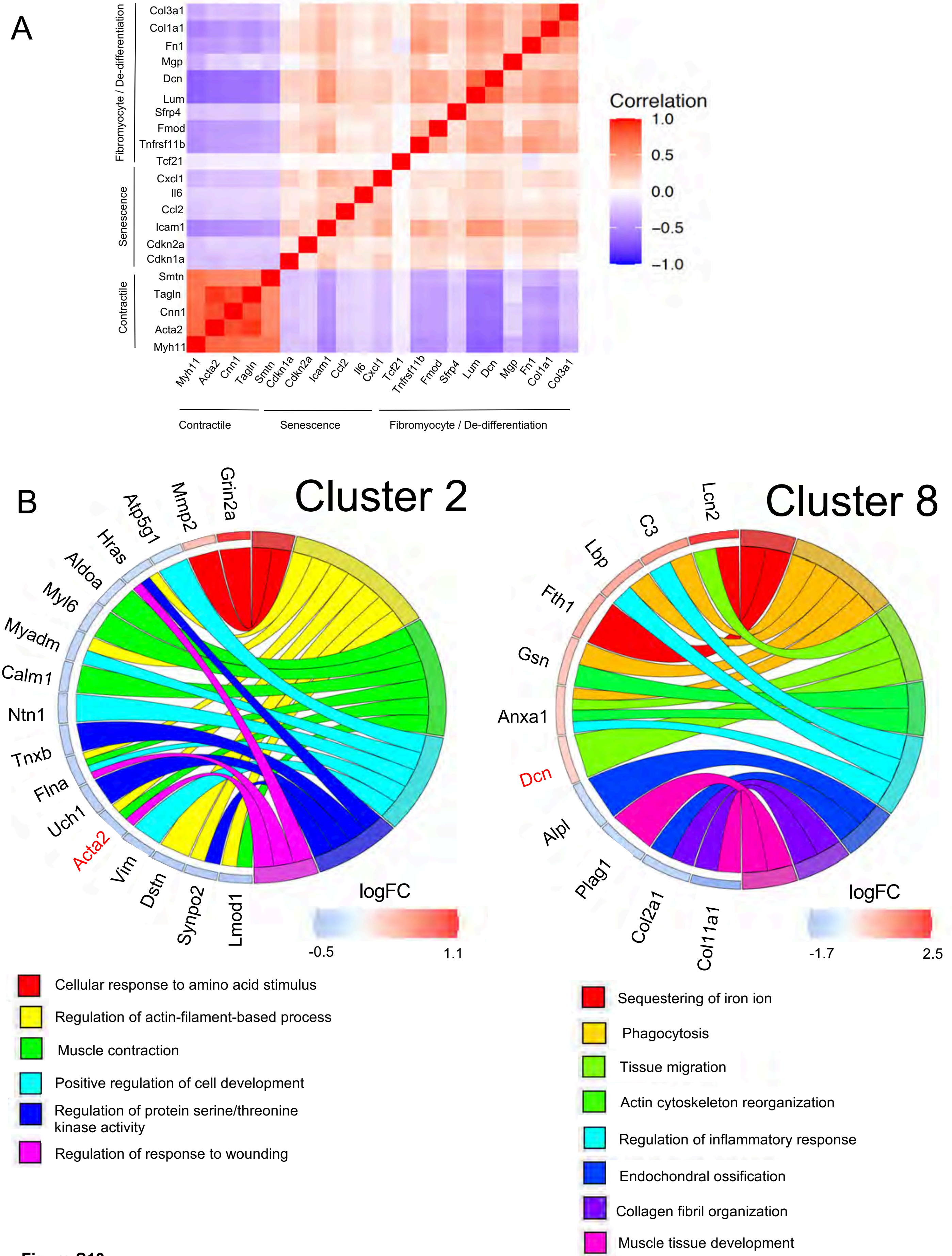

Figure S10

(A) Correlation heatmap of contractile, senescence and fibromyocyte / de-differentiation markers in mouse atherosclerotic plaques from *Myh11-Cre^{ERt2}/Confetti/ApoE$^{-/-}$* (*ApoE$^{-/-}$*) and *Myh11-Cre^{ERt2}/Confetti/Sm22a-Trf2^{T188A}/ApoE$^{-/-}$* (*Trf2^{T188A}/ApoE$^{-/-}$*) mice after high fat feeding. **(B)** Chord plots for selected GO terms enriched within the upregulated or downregulated genes in *Myh11-Cre^{ERt2}/Confetti/Sm22a-Trf2^{T188A}/ApoE$^{-/-}$* (*Trf2^{T188A}/ApoE$^{-/-}$*) *vs. Myh11-Cre^{ERt2}/Confetti/ApoE$^{-/-}$* (*ApoE$^{-/-}$*) mouse plaque VSMCs in Clusters 2 and 8 after high fat feeding.

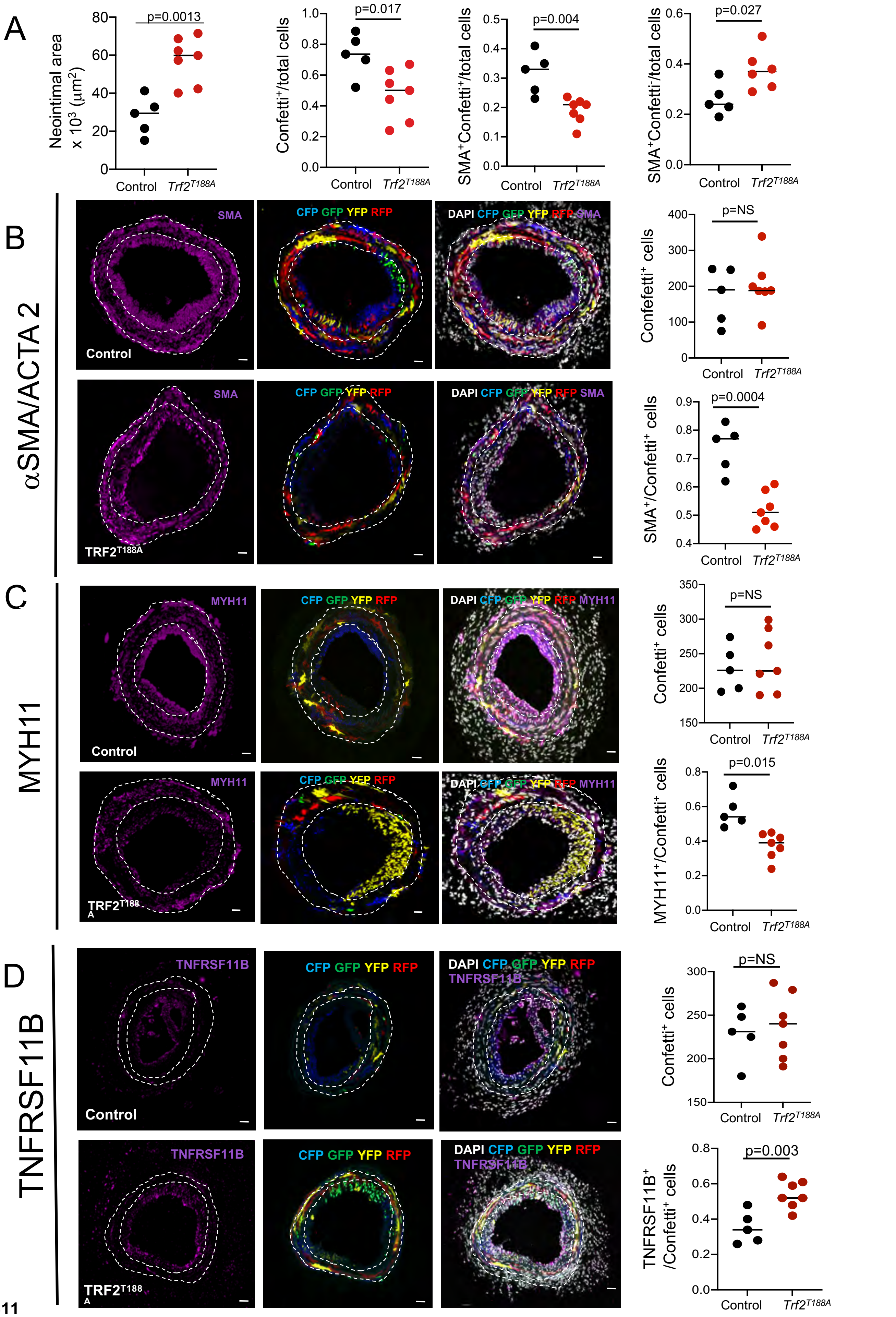

Figure S11

(A) Neointimal area and proportion of Confetti+/Total cells, SMA+/Confetti+/Total cells and SMA-/Confetti-/Total cells in *Trf2^{T188A}* and control mice **(B-D)** Left common carotid arteries of *Trf2^{T188A}* and control mice 28d post ligation (n=4-5), showing immunofluorescence signal for αSMA/ACTA2 **(A),** MYH11 **(B)** or TNFRSF11B **(C)**(left panels), the *Confetti* reporter (red, blue, green, yellow)(middle panels), or these signals combined with DAPI (white)(right panels). Scale bar=30µm. Dashed lines indicate EEL and IEL. Graphs of Confetti+ neointimal cells or marker+/Confetti ratio for control of TRF2T188A mice (n=5-7). Unpaired t test.

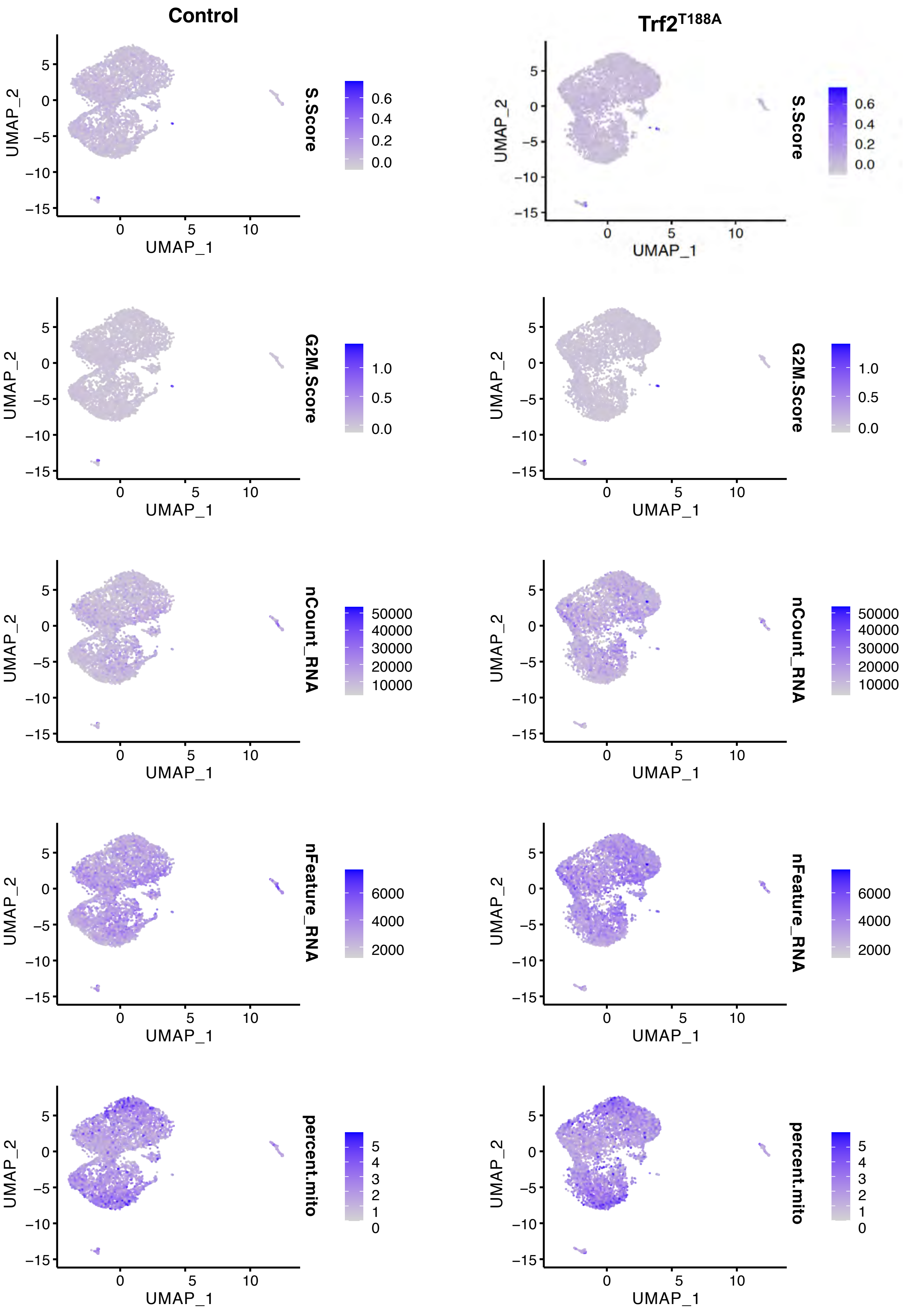

Figure S12
UMAPs showing S-phase score, G2-M phase score, number of unique molecular Identifiers (UMIs, nCount_RNA), number of genes (nFeatures), and percent mitochondrial genes for VSMCs isolated after carotid ligation from control or Trf2^{T188A} mice.

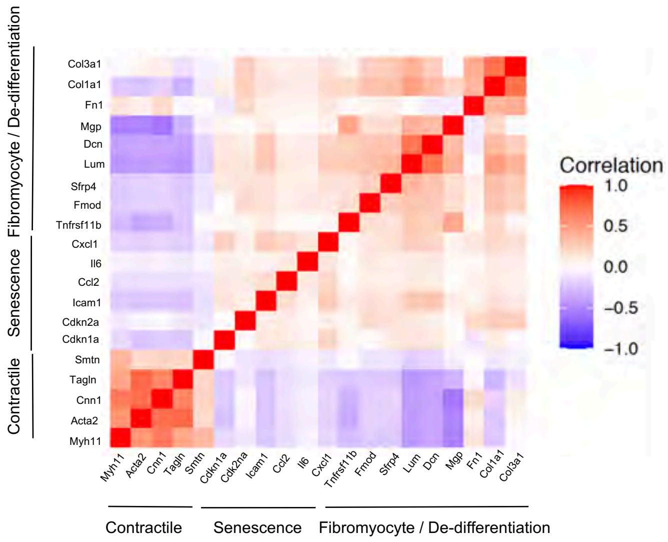

Figure S13
Correlation heatmap of contractile, senescence and fibromyocyte / de-differentiation markers in *Sm22a-Trf2*^{T188A} and wild-type littermate control mice after carotid artery ligation.

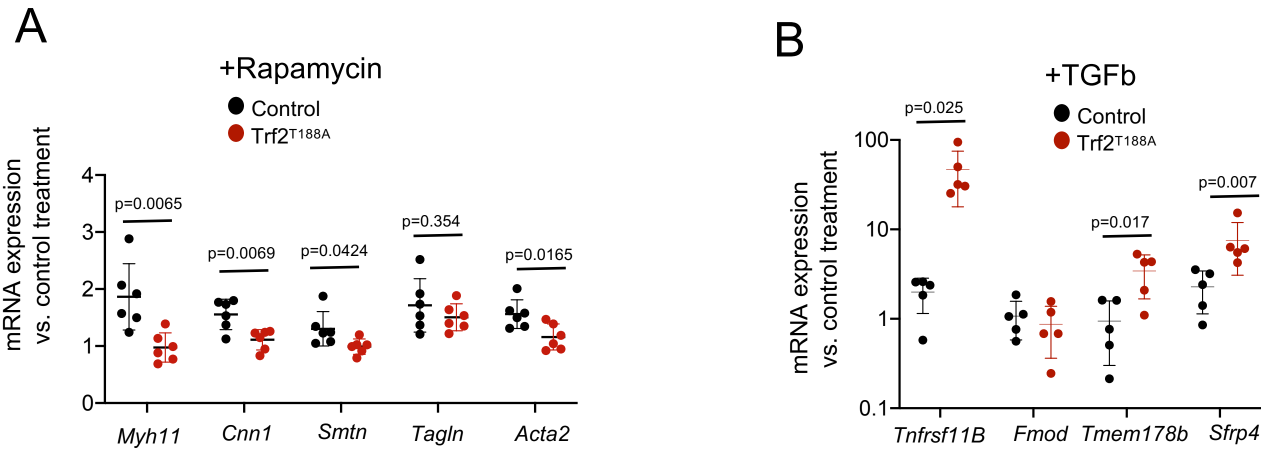

Figure S14
(A) mRNA expression of contractile genes in cultured mVSMCs derived from control (wild-type) or *Trf2*^{T188A} mice treated with 50nM Rapamycin for 24h vs. control treatment. **(B)** mRNA expression of de-differentiation genes in cultured mVSMCs derived from control (wild-type) or *Trf2*^{T188A} mice treated with TGFb for 48 hours vs. control treatment. n=5-6, unpaired t-test.

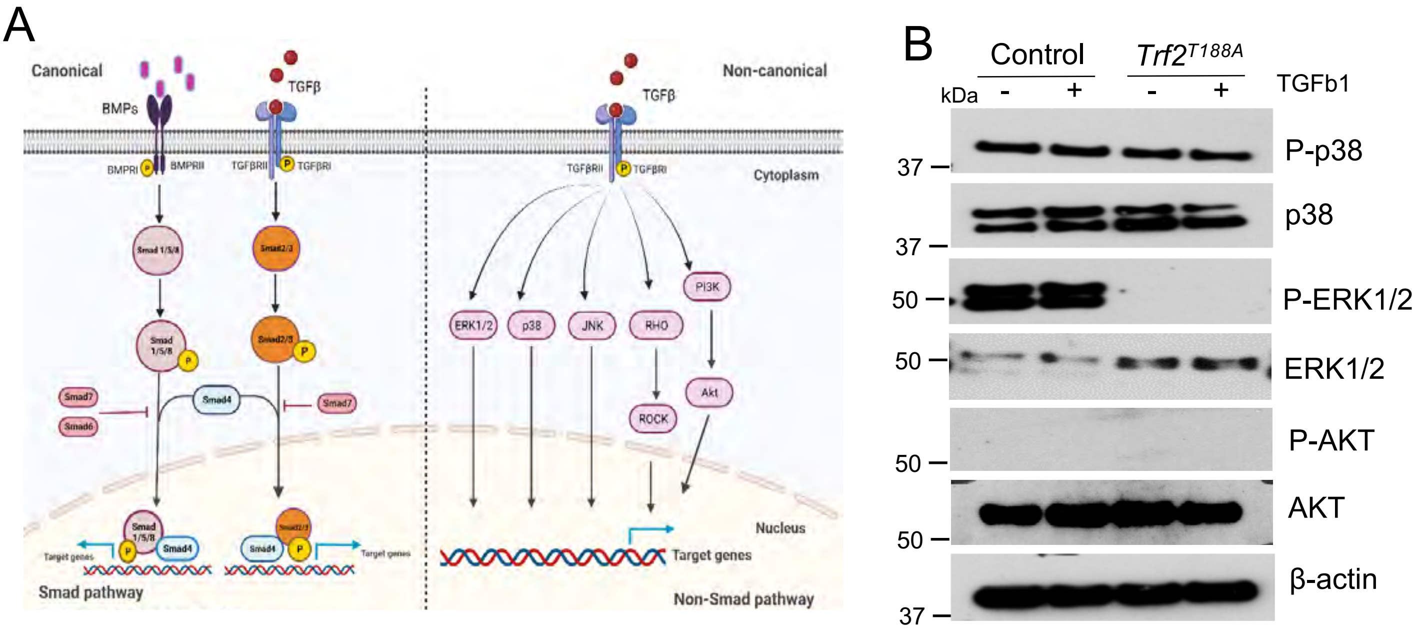

Figure S15
A-B Schematic of canonical and non-canonical Tgfb signalling in VSMCs **(A).** Western blot for non- canonical Tgfb signalling pathways in Control and *Trf2*^{T188A} cells with or without TGFb treatment **(B)**.

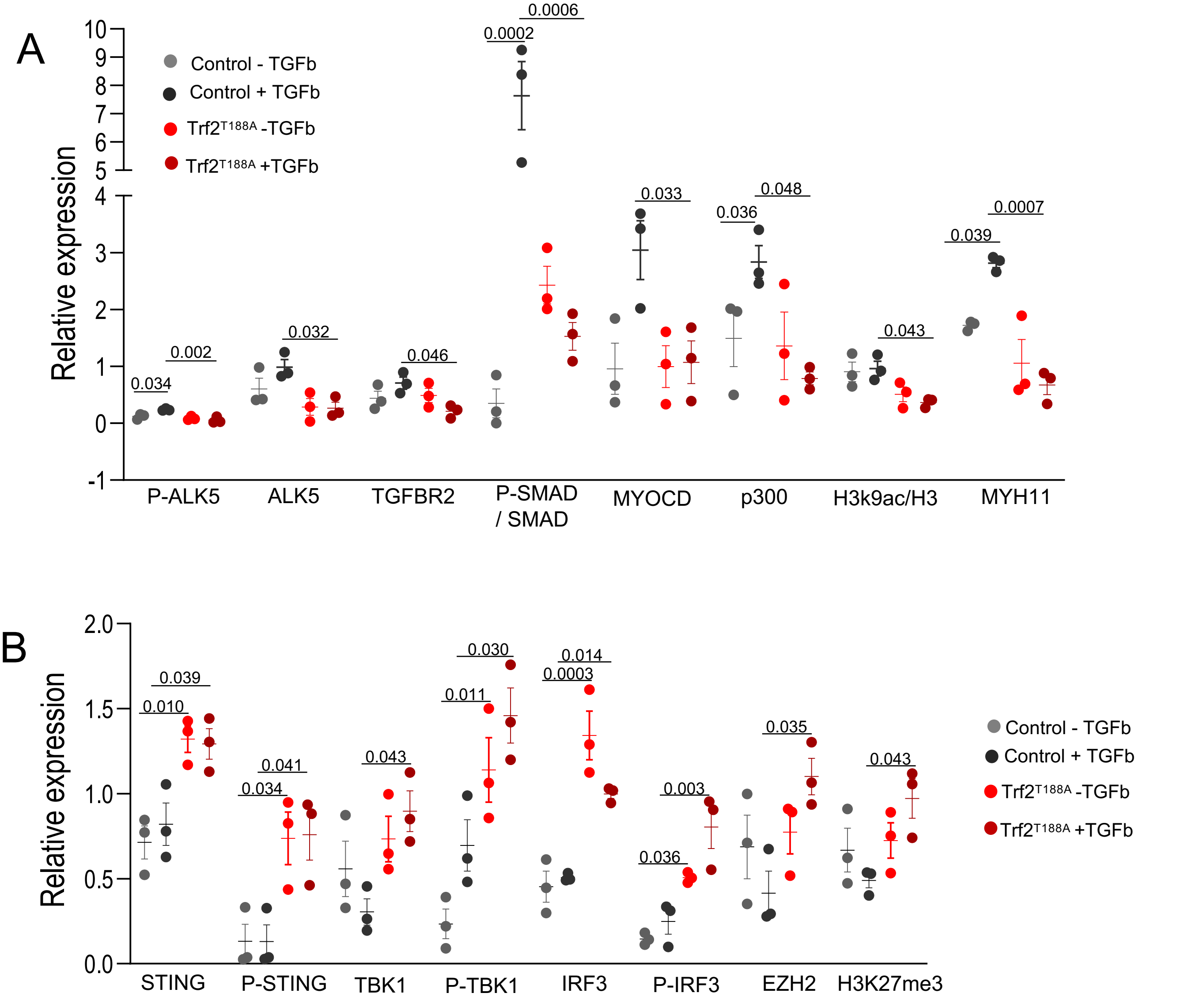

Figure S16

(A-B) Western blot quantification for Tgfb signalling proteins **(A)** or Sting signalling proteins **(B)** in control or *Trf2*^{T188A} VSMCs with or without TGFb treatment**.** (n=3). 1-way ANOVA.

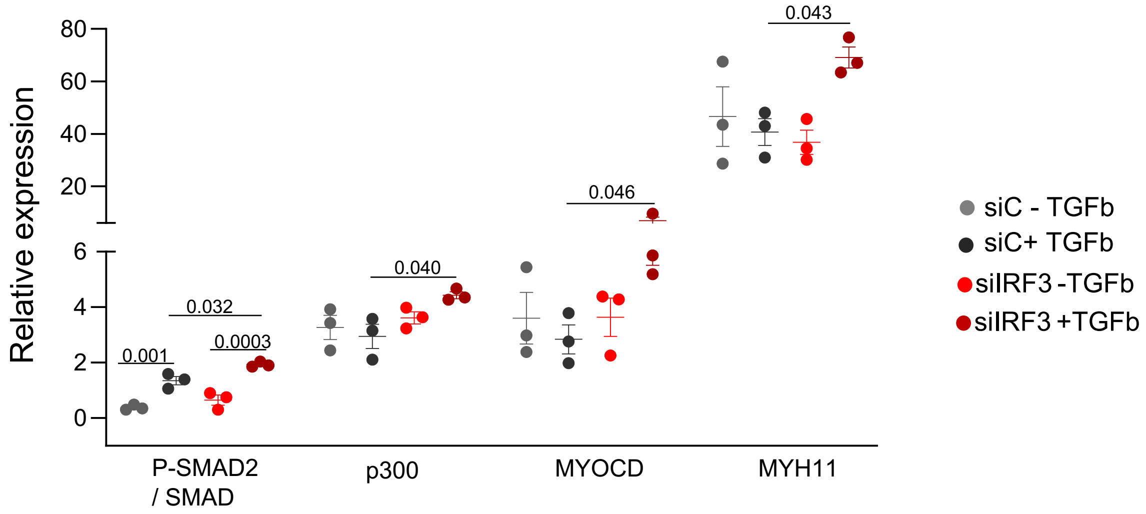

Figure S17

Western blot quantification for p-SMAD/SMAD2, p300, MYOCD and MYH11 after *Irf3* (siIRF3) or control siRNA (siC) treatment of *Trf2*^{T188A} VSMCs, with or without TGFb treatment (n=3). 1-way ANOVA.

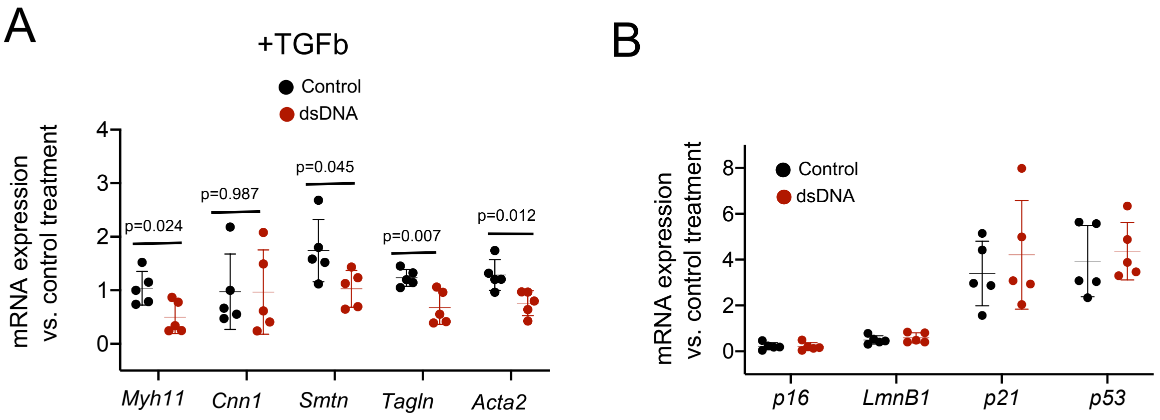

Figure S18
(A) mRNA expression of contractile markers in control mouse VSMCs transfected with exogenous dsDNA vs. vehicle control followed by 48hrs 10ng/ml TGFb**. (B)** mRNA expression of senescence markers (p16 and Lamin B1), or DNA damage markers (p21 and p53) in control mouse VSMCs transfected with exogenous dsDNA vs. vehicle control. Unpaired t-test, n=5.
