# Supplementary material for: Premature cell senescence promotes vascular smooth muscle cell phenotypic modulation and resistance to re-differentiation
Source: Cardiovasc Res. 2025 Jun 10;121(9):1448–63. doi: 10.1093/cvr/cvaf102 (PMC12352304; doi:10.1093/cvr/cvaf102)
Supplement: cvaf102_Supplementary_Data [file cvaf102_supplementary_data.zip › Unedited gels 4.pdf]

Fig.3E

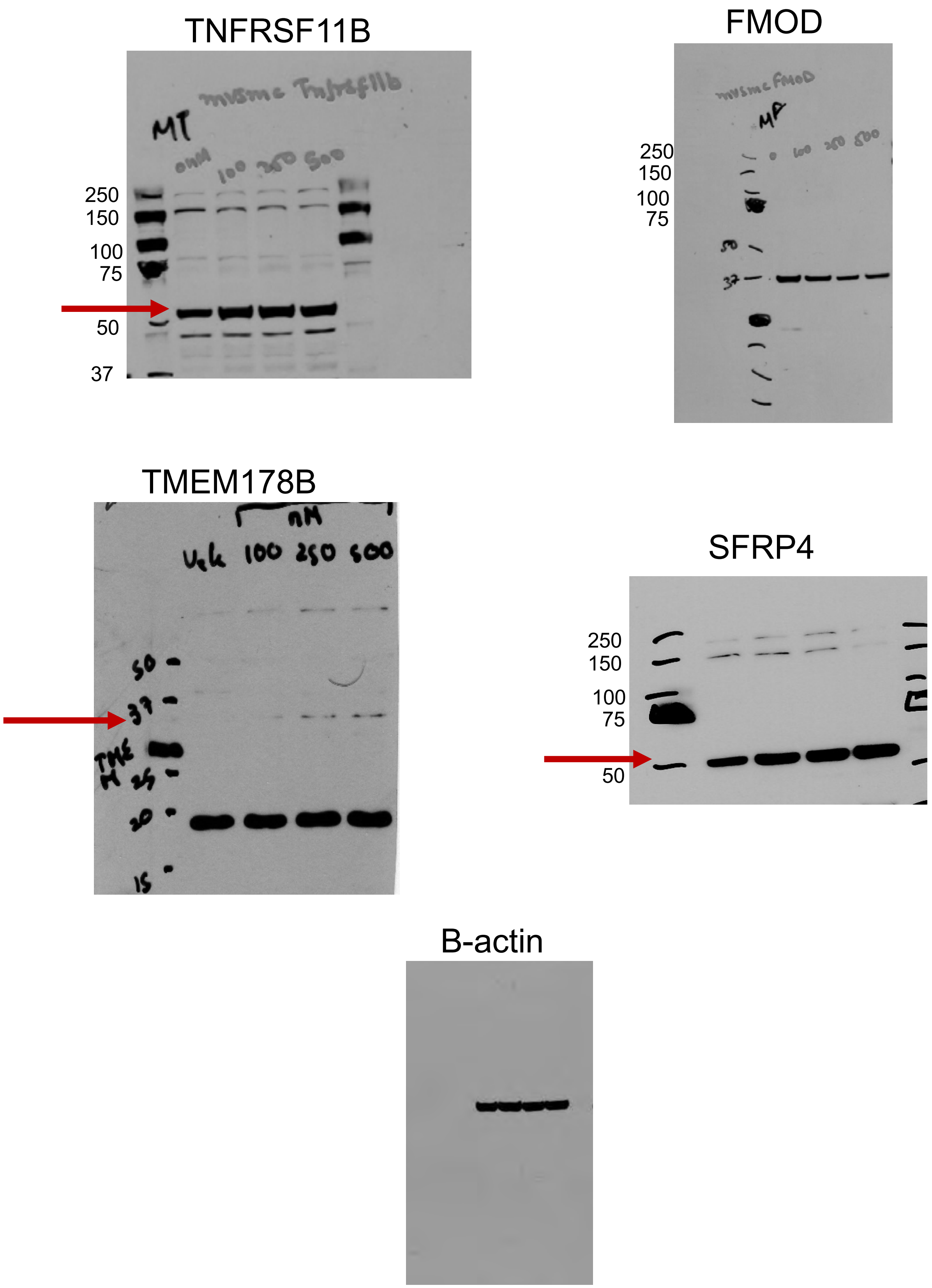

TNFRSF11B
250
150
100
75
50
37
FMOD
250
150
100
75
TMEM178B
SFRP4
250
150
100
75
50
B-actin

Fig.7E

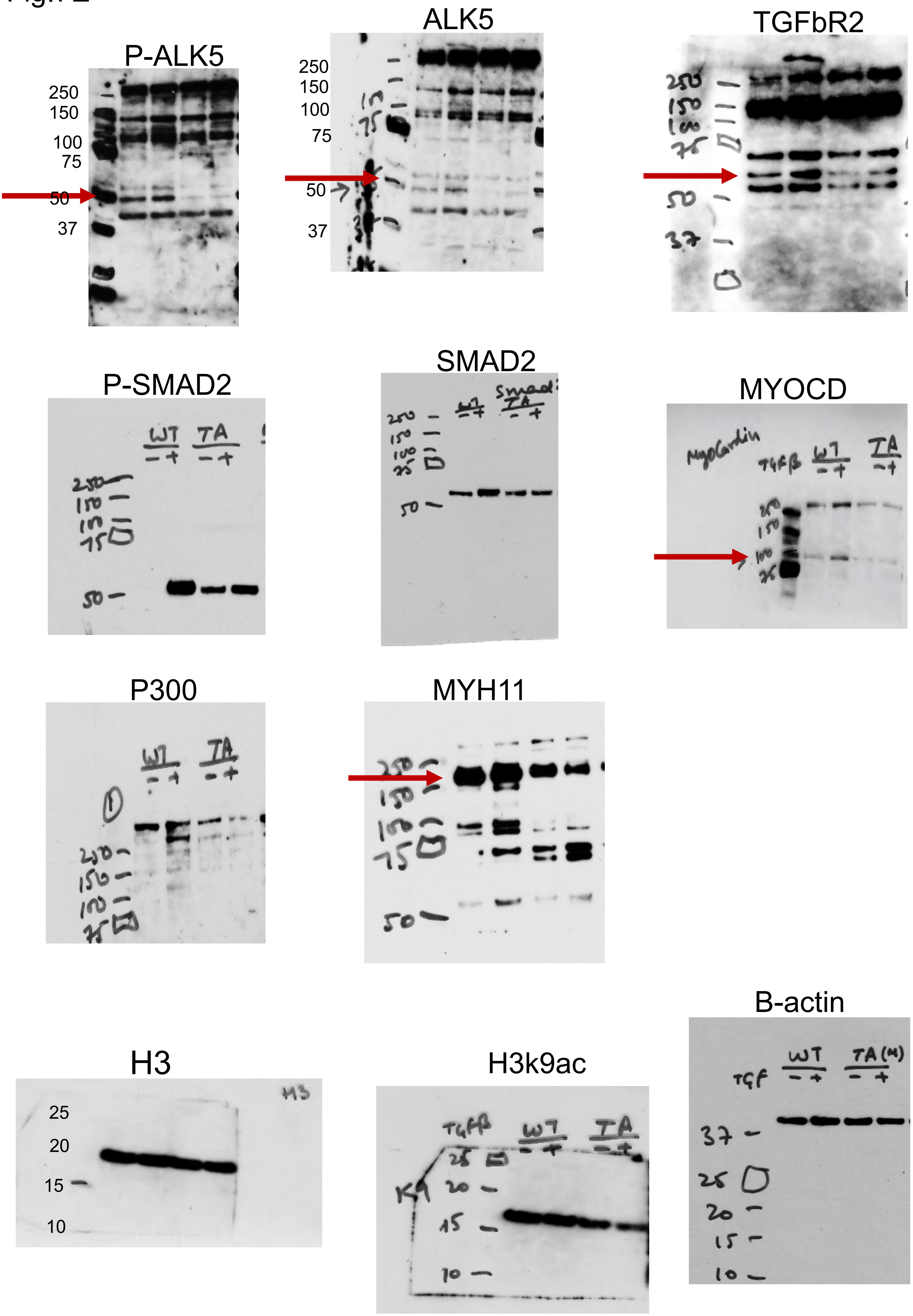

P-ALK5
250
150
100
75
50
37
ALK5
250
150
100
75
50
37
TGFbR2
P-SMAD2
SMAD2
MYOCD
P300
MYH11
H3
25
20
15
10
H3k9ac
B-actin

Fig.7I

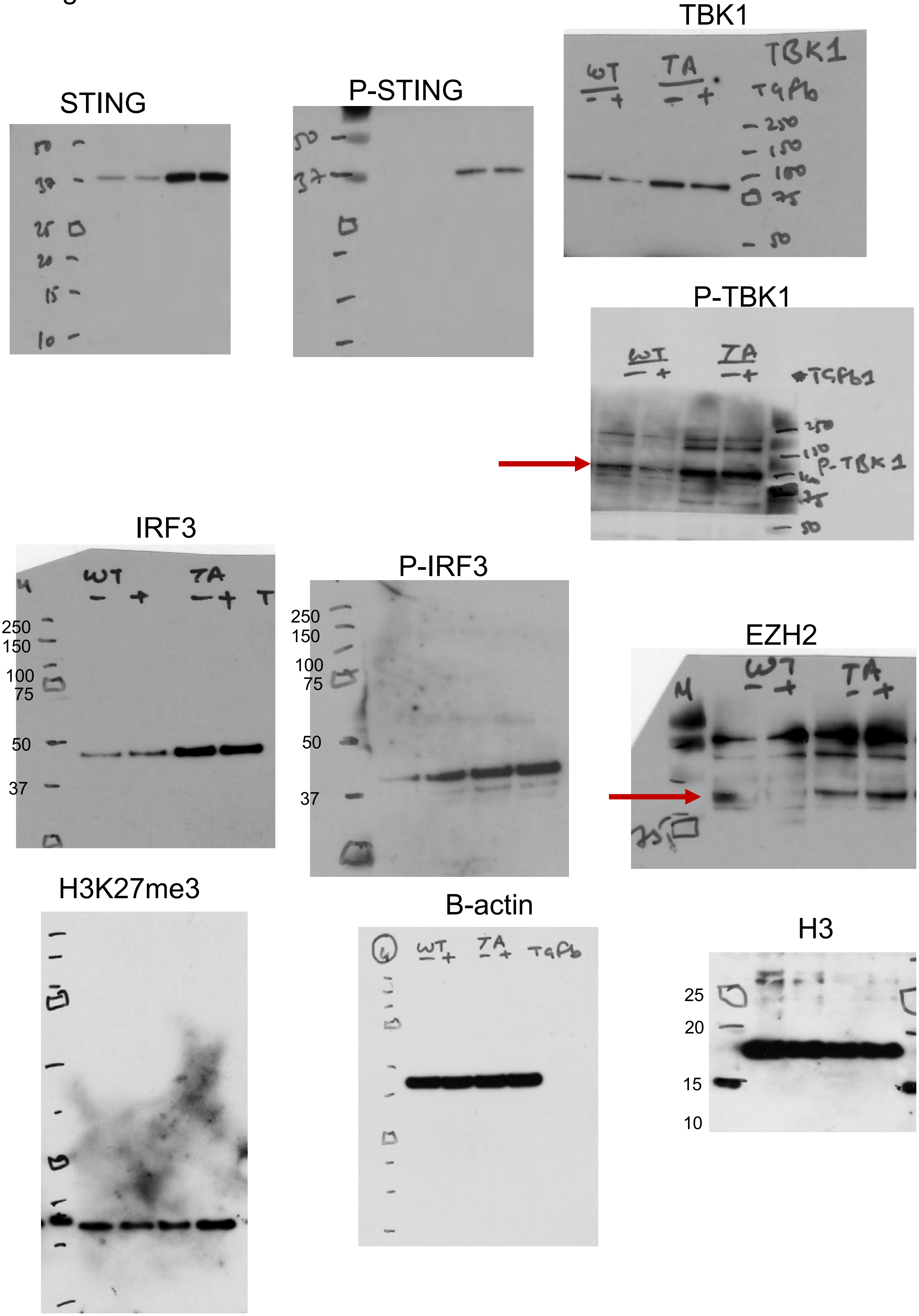

TBK1
STING
P-STING
P-TBK1
IRF3
P-IRF3
EZH2
250
150
100
75
50
37
H3K27me3
B-actin
H3
25
20
15
10

Fig.7K

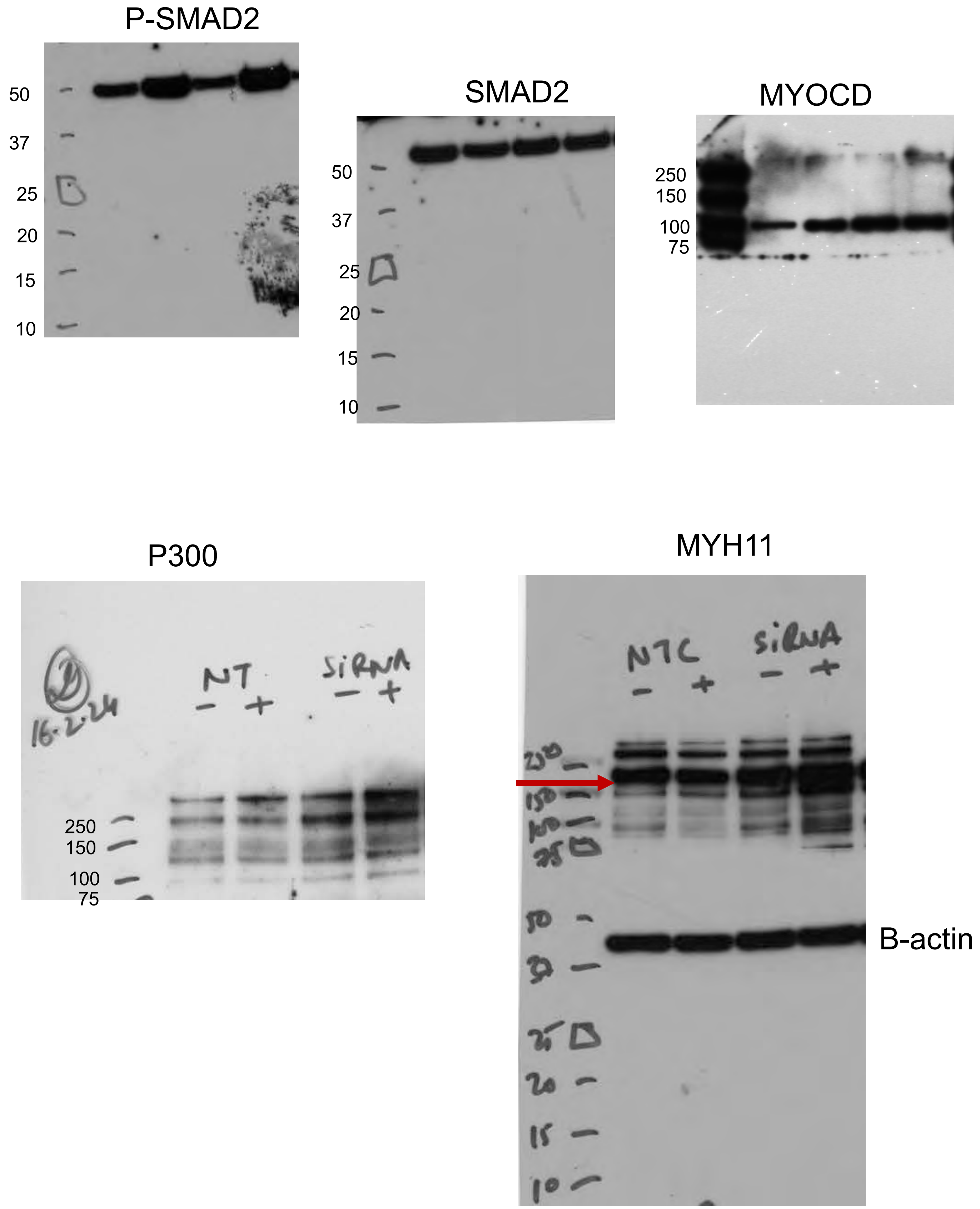
P-SMAD2
50
37
25
20
15
10
SMAD2
50
37
25
20
15
10
MYOCD
250
150
100
75
P300
16.2.24
NT
- +
SiRNA
- +
250
150
100
75
MYH11
NTC
- +
SiRNA
- +
B-actin

Supplemental Figures

Fig.S1 A

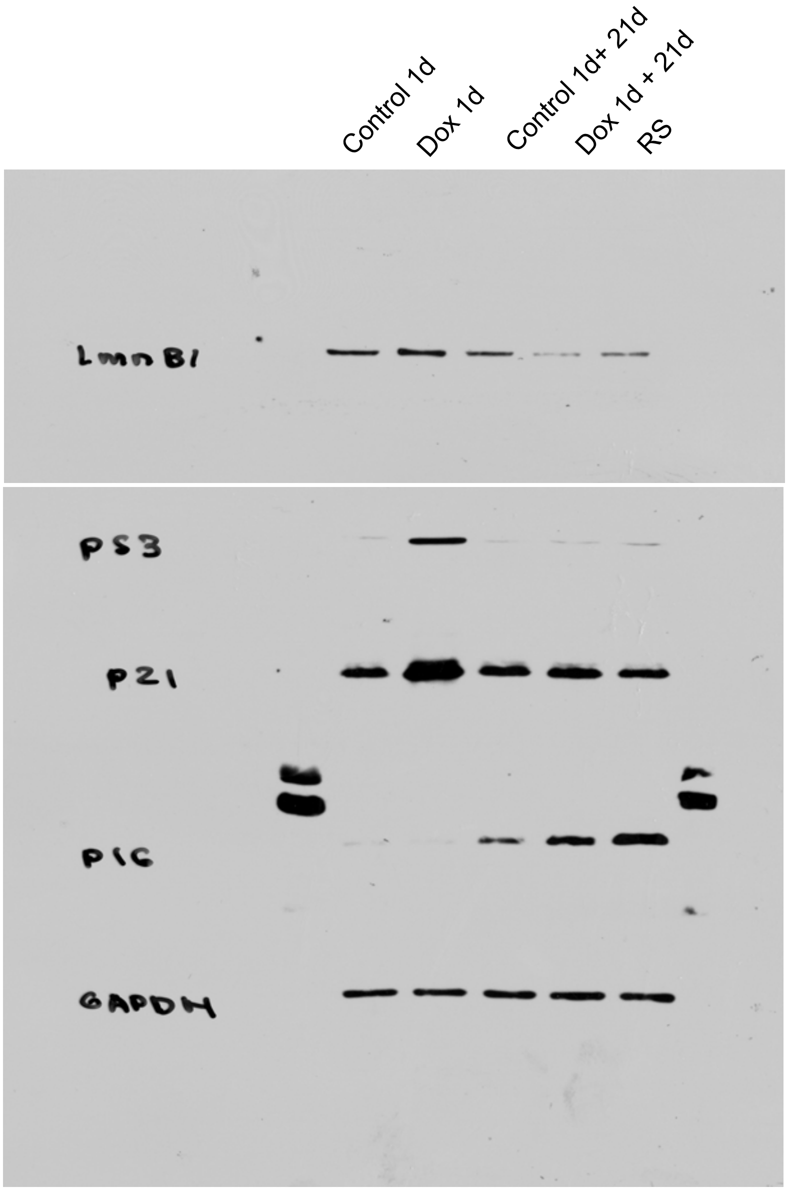

Fig.S3

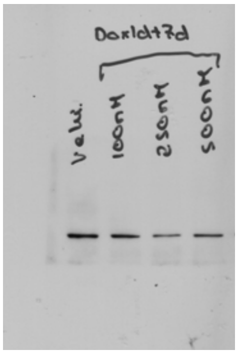

Lamin B1

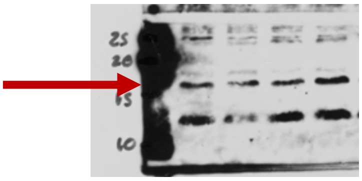

P16

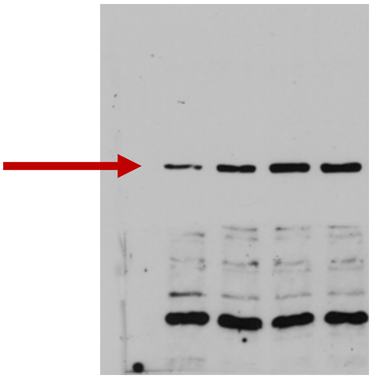

P53

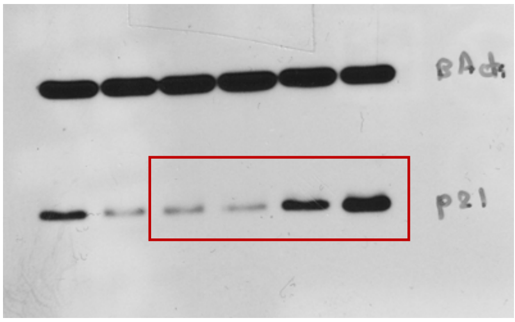

P21

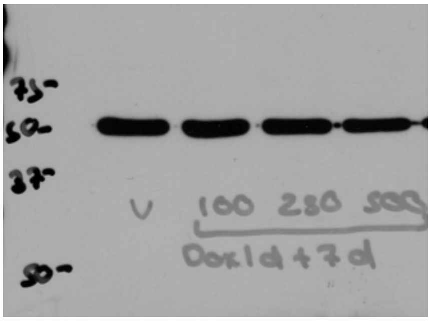

α/β Tubulin

Fig.S15

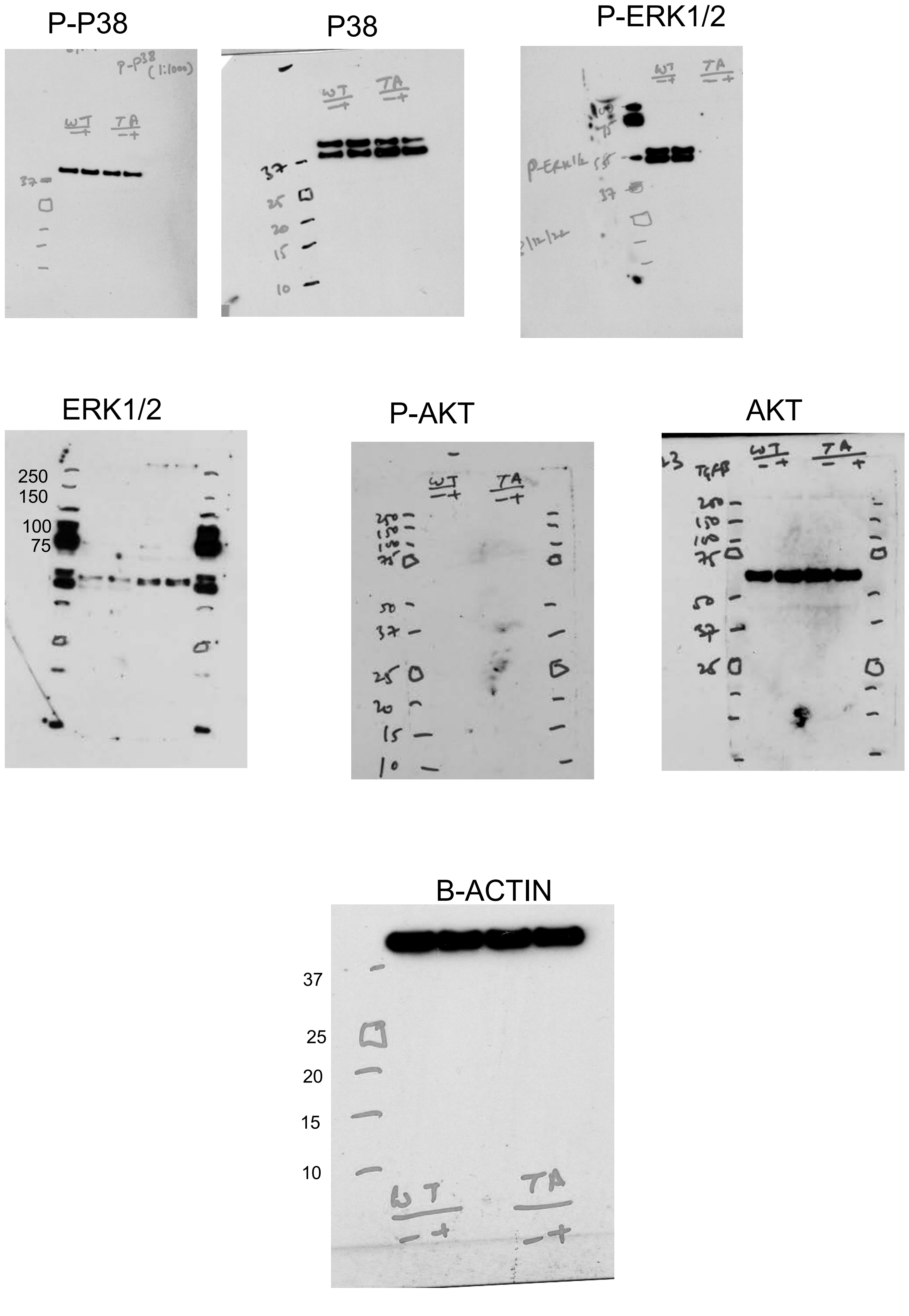

P-P38
P38
P-ERK1/2
ERK1/2
250
150
100
75
P-AKT
AKT
B-ACTIN
37
25
20
15
10
